# PDSE-Lite: lightweight framework for plant disease severity estimation based on Convolutional Autoencoder and Few-Shot Learning

**DOI:** 10.3389/fpls.2023.1319894

**Published:** 2024-01-08

**Authors:** Punam Bedi, Pushkar Gole, Sudeep Marwaha

**Affiliations:** ^1^ Department of Computer Science, University of Delhi, New Delhi, India; ^2^ ICAR-Indian Agricultural Statistics Research Institute, New Delhi, India

**Keywords:** Convolutional Autoencoder, few-shot learning, deep learning, automatic plant disease severity estimation, AI in agriculture

## Abstract

Plant disease diagnosis with estimation of disease severity at early stages still remains a significant research challenge in agriculture. It is helpful in diagnosing plant diseases at the earliest so that timely action can be taken for curing the disease. Existing studies often rely on labor-intensive manually annotated large datasets for disease severity estimation. In order to conquer this problem, a lightweight framework named “PDSE-Lite” based on Convolutional Autoencoder (CAE) and Few-Shot Learning (FSL) is proposed in this manuscript for plant disease severity estimation with few training instances. The PDSE-Lite framework is designed and developed in two stages. In first stage, a lightweight CAE model is built and trained to reconstruct leaf images from original leaf images with minimal reconstruction loss. In subsequent stage, pretrained layers of the CAE model built in the first stage are utilized to develop the image classification and segmentation models, which are then trained using FSL. By leveraging FSL, the proposed framework requires only a few annotated instances for training, which significantly reduces the human efforts required for data annotation. Disease severity is then calculated by determining the percentage of diseased leaf pixels obtained through segmentation out of the total leaf pixels. The PDSE-Lite framework’s performance is evaluated on Apple-Tree-Leaf-Disease-Segmentation (ATLDS) dataset. However, the proposed framework can identify any plant disease and quantify the severity of identified diseases. Experimental results reveal that the PDSE-Lite framework can accurately detect healthy and four types of apple tree diseases as well as precisely segment the diseased area from leaf images by using only two training samples from each class of the ATLDS dataset. Furthermore, the PDSE-Lite framework’s performance is compared with existing state-of-the-art techniques, and it is found that this framework outperformed these approaches. The proposed framework’s applicability is further verified by statistical hypothesis testing using Student t-test. The results obtained from this test confirm that the proposed framework can precisely estimate the plant disease severity with a confidence interval of 99%. Hence, by reducing the reliance on large-scale manual data annotation, the proposed framework offers a promising solution for early-stage plant disease diagnosis and severity estimation.

## 1 Introduction

The agricultural sector can potentially impact the economies of various agrarian countries. In India, the agriculture sector contributes around 18.3% of the country’s Gross Domestic Product, and more than 50% workforce is engaged in agriculture or allied fields (Ministry of Statistics & Programme Implementation, 2023). Furthermore, the agricultural sector’s growth is also essential to fulfill the world’s food demand, which has been increasing exponentially in the past few decades. The growth of the agricultural sector is hindered by many hurdles, and as a result, a sustainable food grain production system is continually being developed by agrarian scientists. Early-stage plant disease detection with its severity estimation is a major challenge in front of agrarian researchers as it hampers both food grain quality and quantity. Moreover, plant disease severity estimation is also necessary for tracking plant diseases and treatment planning. Conventionally, farmers and agricultural scientists do the manual examination of plant leaves to detect the probable disease and then estimate disease severity with their expertise. As a result of technical developments in the computer vision domain, nowadays, plant disease detection and severity estimation, is being done by using computational intelligence techniques and digital leaf images. In computer vision, the problem of detecting plant diseases can be viewed as an image classification task, wherein a Machine Learning (ML) or Deep Learning (DL) model is trained to categorize leaf images as either healthy or diseased based on their visual characteristics. The process of plant disease severity estimation via digital leaf images can be conceptualized in two ways. In the first scenario, plant leaf images can be categorized in various severity scales with the help of plant pathologists. Then, these images can be classified by designing any image classification model based on ML or DL. In the second case, this problem is solved in two steps; initially, the diseased regions are identified by segmenting the corresponding pixels in the leaf image via image segmentation. Subsequently, the disease severity is computed via calculating percentage of diseased pixels out of total leaf pixels.

Nowadays, DL-based models are widely used by researchers for automatic plant disease recognition and severity estimation. However, limited research works are available in the literature on plant disease severity estimation compared to plant disease detection. The existing research works focused on plant disease severity estimation are divided into four broad groups. First group of research works applied various Digital Image Processing (DIP) techniques like image thresholding, Otsu segmentation, etc., to segment out the diseased area from leaf images (Bock et al., 2010; Patil and Bodhe, 2011; Barbedo, 2014; Dhingra et al., 2018). Although these DIP methods can segment the diseased areas from leaf images, but their performance significantly decreases when applied to leaf images captured from the real field with complex backgrounds. Second category of research works done for segmenting diseased areas from plant leaf images is based on ML techniques like Fuzzy C-Means clustering, K-Means clustering, etc., (Biswas et al., 2014; Mwebaze and Owomugisha, 2016; Sethy et al., 2018). Though the results achieved via ML techniques are much better than the DIP techniques, but they suffer from some major drawbacks. K-means clustering is sensitive to hyperparameter initialization, leading to variable segmentation outcomes. Furthermore, Fuzzy C-Means clustering faces high computational complexity and dependence on the fuzziness parameter, requiring careful parameter selection for accurate results. The third category of research works has used DL techniques for plant disease severity identification by classifying the leaf image into one of the severity level classes (Wang et al., 2017; Haque et al., 2022a). Though these works can identify various severity levels of plant disease, but they are unable to quantify the severity of plant disease in percentage. Moreover, classifying leaf images into predefined severity level classes instead of evaluating the severity percentage has various drawbacks, such as limited granularity, subjective interpretation, loss of information, and the inability to track disease changes over time. Assessing the severity percentage provides more detailed information for informed decision-making in plant health management. The fourth type of research works has leveraged various DL segmentation techniques for segmenting the diseased area from leaf images for quantifying plant disease severity in percentage (Chen et al., 2021; Wang et al., 2021a; Pal and Kumar, 2023). However, training these models requires a large amount of annotated leaf images for precise segmentation of disease areas from leaf images, and in the real world, creating such datasets is a very laborious task. Furthermore, training any DL model with a limited amount of annotated leaf images would result in model overfitting. Various researchers have primarily used two types of data augmentation techniques, namely, Digital Image Processing-based techniques (Chohan et al., 2020; Haque et al., 2022b) and Generative Adversarial Networks (Abbas et al., 2021; Zhang et al., 2022) to conquer this problem of limited annotated data. Though these data augmentation techniques can generate an adequate amount of leaf images along with their annotations, but the performance of any model trained on these images drastically decreases when deployed in the real world. Therefore, the advantages of Few Shot Learning, which uses few instances for training, can be leveraged to develop a Machine Intelligence model for plant disease severity estimation.

Conventional ML and DL techniques necessitate a huge annotated dataset for better generalization. However, in the real world, acquiring abundant labeled data requires a lot of human effort. Therefore, FSL techniques are utilized in this research work to conquer this drawback of ML or DL models by using limited labeled data for training. The FSL techniques are based on Meta-Learning or Learning to Learn approaches. These techniques draw inspiration from human developmental theory, which emphasizes the acquisition of priors from past experiences to enhance the effectiveness of learning new tasks. For example, a traditional ML or DL model only tackles individual classification tasks, whereas a Meta-learning-based ML or DL model comprehends the process of acquiring skills for solving classification tasks through exposure to numerous analogous tasks. Hence, when the Meta-learning-based ML or DL model tries to work on a similar but new task, then it can solve the new task quickly and better than a traditional ML or DL model, which has no prior experience in solving this task (Yang et al., 2020). This motivated different researchers to solve many real-world problems by utilizing FSL approaches which require a limited amount of data for training (Wang et al., 2021b; Kumar and Mishra, 2023). Various researchers have also leveraged FSL in the agricultural sector for plant disease recognition with severity estimation (Argüeso et al., 2020; Liang, 2021; Tassis and Krohling, 2022). However, most of these works are either focused only on plant disease detection or identifying plant disease severity by classifying the leaf image into one of the several predefined severity level classes. To the best of our information, none of the existing research works quantify the severity level of plant diseases between 0% to 100% by utilizing few training instances. Hence, in order to bridge this research gap, a lightweight framework named “PDSE-Lite” based on CAE and FSL is proposed in this manuscript for plant disease detection and severity quantification. The major contributions of this paper have been listed below:

• A few-shot and lightweight framework named “PDSE-Lite” based on CAE and FSL is designed and developed to detect disease presence within leaf images accurately and then precisely segment the diseased pixels for quantifying the plant disease severity in the range of 0% to 100% by using only a few annotated leaf images for training.
• The PDSE-Lite framework has been trained and tested on an in-field Apple leaf disease dataset (Feng and Chao, 2022) to showcase its pertinence in real-world scenarios. Additionally, the comparison of the proposed framework is done with the existing state-of-the-art models for plant disease recognition and segmentation of diseased areas from leaf images.

The remainder of the paper comprises six sections. Section 2 delves into the pertinent literature related to this research work. Section 3 describes the PDSE-Lite framework, while Section 4 describes the experimentation done in this research. Section 5 provides the results obtained from experimentation, which are further analyzed and discussed in Section 6. Lastly, in Section 7, the conclusion and future perspectives of this research work have been given.

## 2 Related work

Numerous research works have been done in recent years for automatically diagnosing plant diseases via Machine Intelligence and digital images of plant leaves. The plant disease diagnosis process typically involves two steps: disease detection and severity identification. While there are ample research works available on disease detection, the literature addressing the quantification of plant disease severity is relatively limited. In this section, firstly, the research works focused on plant disease detection are given in subsection 2.1. Subsequently, the research efforts undertaken for plant disease severity estimation are discussed in subsection 2.2.

### 2.1 Research work focused on plant disease detection

The research pertaining to plant disease recognition is categorized into two groups based on the number of diseases they can identify. First group of research works is based on binary classification, i.e., only identifying whether the leaf image is healthy or diseased. For example, Bedi and Gole (2021a) utilized a combination of CNN and CAE for diagnosing bacterial spot disease in peach plants. In their research work, they achieved 98.38% using only 9914 trainable weight parameters. Another study conducted by Chowdhury et al. (2021) employed EfficientNetB7 model for recognizing diseases in tomato plants. According to their findings, this CNN architecture achieved a testing accuracy of 99.95 ± 0.03 with 95% confidence interval for identifying diseased tomato plant leaves. Kukreja et al. (2021) built a custom CNN model to diagnose disease in potato plants. Their proposed CNN model achieved a testing accuracy of 90.77% in detecting diseased potato plant leaves. In another work, Bedi and Gole (2021b) proposed a novel PlantGhostNet model encompassing Squeeze-and-Excitation and Ghost modules for diagnosing bacterial spot disease of peach plants. Their PlantGhostNet model detected the peach plant’s bacterial sport disease with 99.51% testing accuracy. Classifying plant leaf images to either diseased or healthy class without identifying the specific disease hampers appropriate treatment selection, targeted control measures, understanding disease dynamics, and precise monitoring and tracking. Therefore, researchers are actively developing machine intelligence models that not only identify the presence of disease but also focus on accurately identifying diseases affecting plants. Nigam et al. (2023) evaluated the performances of eight variants of EfficientNet model to diagnose diseases in wheat plants. The authors of this paper concluded that the EfficientNet-B4 variant achieved highest accuracy among other variants, i.e., 99.35%. The research work done by Shewale and Daruwala (2023) proposed a novel custom CNN architecture for identifying nine diseases of tomato plants. As reported by the authors, their model achieved higher accuracy than other predefined CNN architectures with 99.81% accuracy. Gole et al. (2023) proposed a modified lightweight Vision Transformer (ViT) model named “TrIncNet” to identify plant diseases. They evaluated the TrIncNet model’s performance on Maize dataset (comprising in-field healthy and diseased leaf images of Maize plants) and PlantVillage dataset. As per their paper, the TrIncNet model achieved 96.93% and 99.93% testing accuracy on Maize and PlantVillage datasets, respectively.

Furthermore, the research works present in the literature are majorly divided into two categories based on the technology used to build Machine Intelligence models for plant disease detection. First type of research works are based on ML models like K-Nearest Neighbor (KNN), Decision Tree, etc. Ramesh et al. (2018) employed the Histogram of an Oriented Gradient algorithm to extract various important features from leaf images. Then, they trained various ML algorithms like Naïve Bayes classifier, Logistic Regression, etc., with these extracted features to identify plant diseases accurately. According to their paper, the Random Forest classifier outperformed others with 70.14% accuracy. In another research work, Tulshan and Raul (2019) designed and developed an ML-based framework for automatically recognizing plant diseases. Their proposed framework first extracts essential features by computing Gray-Level-Cooccurrence Matrix (GLCM) from leaf images. Thereafter, they applied the KNN classifier for identifying plant diseases. Sujatha et al. (2021) analyzed the potential of various traditional ML techniques and CNN models in detecting plant disease by their leaf images. As per their paper, the VGG-19 CNN model outperformed other ML methods with a testing accuracy of 89.5%. This outperforming nature of CNN models over traditional ML models can be argued on the fact that the ML techniques have two major shortcomings. Firstly, they cannot extract different spatial features automatically from leaf images, and secondly, they are not developed in a way that they can use Graphic Processing Units (GPUs) for faster training. These drawbacks of ML techniques are conquered through DL techniques. Therefore, various researchers tried to use DL techniques, particularly CNN, for automatically detecting plant diseases via their digital leaf images. For example, Bedi et al. (2021) experimented with five popular predefined CNN architectures (AlexNet, LeNet5, GoogLeNet, VGG16, and ResNet50) to evaluate their performance in identification of bacterial spot disease of peach plants. As per their paper, the AlexNet CNN model outperformed other models with 98.5% testing accuracy. In another work, Atila et al. (2021) trained an EfficientNet model on the PlantVillage dataset to detect plant diseases, and it achieved 99.91% testing accuracy. Zhao et al. (2023) presented a novel self-supervised contrastive learning-based plant disease detection framework with the advantage of domain adaption. Their proposed method addresses the challenges faced in plant leaf disease identification by using self-supervised learning with large-scale unannotated dataset for pre-training, followed by fine-tuning with domain adaptation. It achieved improved performance by aligning labeled and unlabeled data, resulting in more general visual representations and achieving a high accuracy of 90.52%.

Although the abovementioned research works can accurately detect plant diseases, but all these ML or DL models necessitate huge amount of labeled data to get high accuracy. Nevertheless, in the real world, creating such dataset is very laborious task. Moreover, training these models with limited annotated data can lead to model overfitting problem. In order to address this issue, researchers have predominantly employed two types of data augmentation techniques, namely, image processing-based techniques and GANs. Although these techniques can produce a sufficient number of annotated leaf images, but models trained on such data exhibit significant performance degradation when deployed in the real world. Therefore, various researchers leveraged the advantages of FSL and developed different DL models for plant disease recognition with limited training data. For example, Argüeso et al. (2020) developed an FSL framework based on transfer learning to recognize plant diseases. In order to train their proposed FSL framework, they used the PlantVillage dataset comprising of digital plant leaf images distributed in 38 classes. They divided the PlantVillage dataset into source and target domain, which encompasses of 32 and 6 classes, respectively. They first trained an InceptionV3 CNN model on the source domain and transferred this trained knowledge to learn the features of leaf images present in the target domain. Their proposed model achieved 94% and 91% testing accuracy in target and source domains, respectively. A similar kind of work was also done by Garg and Singh (2023). They trained the MobileNetV2 model and achieved 75.3% accuracy when it was trained with only one image per class in the target domain. Whereas the maximum accuracy claimed in the paper, i.e., 98.17%, is achieved when the model is trained on 100 images per class in the target domain. In the subsequent section, the research works focused on plant disease severity estimation are discussed.

### 2.2 Research work focused on plant disease severity identification

In the literature, researchers have done the plant disease severity estimation using two approaches. In first approach, plant leaf images are classified into predefined severity levels by training any image classification model based on ML or DL techniques. The severity levels are defined with the assistance of plant pathologists. Most of the research works present in the literature based on the first approach have leveraged various predefined CNN models to classify leaf images into various severity levels. For example, Liang et al. (2019) built a PD^2^SE-Net CNN model by combining ShuffleNetV2 and ResNet units for plant disease detection and severity estimation. They trained their proposed model on a manually annotated PlantVillage dataset (plant pathologists manually divided diseased leaf images into general and serious severity classes) and achieved 90.81% accuracy. Similarly, Zhao et al. (2021) also trained their proposed models named SEV-Net on manually annotated PlantVillage dataset for plant disease recognition with severity estimation. The SEV-Net model was built via adding Spatial and Channel Attention blocks in the existing ResNet-50 CNN architecture, and it achieved 95.37% testing accuracy. Some researchers like Haque et al. (2022a), Shu et al. (2023), and Dhiman et al. (2022) trained various state-of-the-art CNN models for classifying plant leaf images into one of the predefined disease severity levels on their own collected in-field leaf images. Verma et al. (2023) established a disease severity estimation framework named for early and late blight diseases in tomato plants. They first captured digital photographs of several infected and healthy tomato plants. Thereafter, these captured leaf images are manually categorized into three severity levels (Early, Middle, Late) with the assistance of agricultural scientists. The MobileNetV2 CNN model was utilized in this research work, and it achieved 94.47% accuracy.

Estimating plant disease severity via first approach, i.e., classifying plant leaf images into predefined severity levels, has various drawbacks, such as limited granularity, subjective interpretation, loss of information, and the inability to track disease changes over time. Thus, in order to conquer these drawbacks, researchers tried to estimate plant disease severity using another approach where plant disease severity is estimated in two steps. In this approach, first, the diseased regions are detected by segmenting the corresponding pixels in the leaf image using image segmentation methods. After that, the disease severity is estimated between 0% and 100% by computing the percentage of diseased pixels out of total leaf pixels. In literature, this approach was followed in several research works like (Wspanialy and Moussa, 2020; Wang et al., 2021a; Ji and Wu, 2022; Divyanth et al., 2023). These research works utilized U-Net and DeepLab-based image segmentation models for segmenting disease pixels from leaf images to further compute disease severity as the percentage of diseased pixels present out of total leaf pixels. In another research work (Pal and Kumar, 2023) built a novel AgriDet model by using the Inception-VGG Network model along with the Kohonen Learning layer for plant disease detection, and it achieved 96% validation accuracy, as claimed in the paper. Furthermore, the Multi-Variate-Grabcut algorithm was also utilized for segmenting the diseased lesions from leaf images. Thereafter, the percentage of diseased pixels out of total leaf pixels was calculated to measure plant disease severity.

Despite of high performance exhibited by aforementioned research works in segmenting diseased areas from leaf images, their training process requires a huge amount of labeled leaf images to precisely segment the disease areas from leaf images. However, in real-world scenarios, creating such datasets is a very challenging and laborious task. Additionally, when training an ML or DL model using limited labeled leaf images, there is a high risk of model overfitting. To address this issue, researchers have predominantly employed two types of data augmentation techniques in their work: image processing-based techniques and Generative Adversarial Networks. These techniques alleviate the problem by artificially generating leaf images with their corresponding segmentation masks. However, the performance of models trained on these artificially generated images significantly decreases when deployed in real-world scenarios. Therefore, various researchers have leveraged the advantages of FSL to develop and train a DL model for plant disease severity quantification only with a few instances for training. For example, (Pan et al., 2022) proposed a two-stage severity estimation framework for leaf scorch disease of strawberry plants using FSL. In the first phase, they utilized the faster RCNN segmentation model to segment strawberry leaves from the captured image, encompassing other objects like mud, plant stems, etc. Afterward, they applied the Siamese Network to classify the leaf images into either healthy, serious scorch, or general scorch severity levels. In order to test their proposed framework on unseen data, they evaluated the model’s performance on 60 new strawberry plant leaf images and claimed that their framework achieved 88.33% accuracy on these images. (Tassis and Krohling, 2022) presented a case study on two FSL techniques, i.e., triplet networks and prototypical networks, which were applied to estimate severity in coffee plant leaves. Moreover, they reported that these FSL techniques achieved 93.25% accuracy in classifying coffee plant leaf images into one of the five severity levels: Very High, High, Low, Very Low, Healthy. Although, (Pan et al., 2022; Tassis and Krohling, 2022) developed a state-of-the-art framework via FSL for estimating the severity of plant diseases, but these research works suffer from a major drawback that they cannot measure the exact amount of disease severity between 0% to 100%. Furthermore, there is still a scope for performance improvement in the aforementioned frameworks, as they have achieved 88.33% and 93.25%, respectively.

Conclusively, it can be observed from the above discussion and **Table 1** that most of the aforementioned research works are either focused on only detecting plant diseases or they estimated plant disease severity via classifying diseased leaf images into one of the several predefined severity levels. Though few works also focus on plant disease severity estimation via computing the percentage of diseased pixels out of total leaf pixels present in any leaf image, but they necessitate a huge amount of labeled leaf images for model training so that it could generalize well on new leaf images. However, annotating huge number of leaf images is a very challenging and laborious task. Thus, the objective of this research work is to design and develop an effective and efficient framework for automatically estimating the severity of plant disease between 0% and 100% using few training samples. Hence, a novel few-shot and lightweight framework named “PDSE-Lite” based on CAE and FSL has been proposed in this research work for diagnosing plant diseases automatically and estimating the severity of identified disease between 0% and 100%. As the proposed framework leveraged the advantages of FSL, and thus, it utilizes only a few training samples for training. Hence, in this way, the proposed framework significantly reduces the human efforts required for annotating leaf images. The next section of this manuscript describes the proposed PDSE-Lite framework.

**Table 1:** Summary of some of the existing research works done for automatic plant disease severity estimation.

Research Work	Technique used	Crop	Performance on test subset of the dataset	Few-Shot (✔/✘)	Disease Detection (✔/✘)	Severity Estimation (✔/✘)	Number of trainable parameters used
Chao et al. (2020)	Xception DenseNet (XDNet)	Apple	98.82% accuracy	✘	✔	✘	10.16 million
Yang et al. (2022)	EfficientNet-MG CNN architecture	Apple	99.11% accuracy	✘	✔	✘	8.42 million
(Ji and Wu, 2022)	DeepLabV3+ based on ResNet50	Grape	93.16% MeanIoU	✘	✔	✔	11.85 million
Firdous et al. (2023)	DenseNet-121	Apple	98.15% accuracy	✘	✔	✘	8.1 million
(Li et al., 2023)	Vision Transformer along with Convolutional Block Attention Module (CBAM) block	Wheat Coffee Rice	94.9% accuracy 87.6% accuracy 92.0% accuracy	✘	✔	✘	5.06 million
(Xing et al., 2023)	PCA-Logistic regression analysis with Mask R-CNN	Apple	90.12% accuracy	✘	✔	✔	63.64 million

## 3 Proposed PDSE-Lite framework

**Figure 1:**
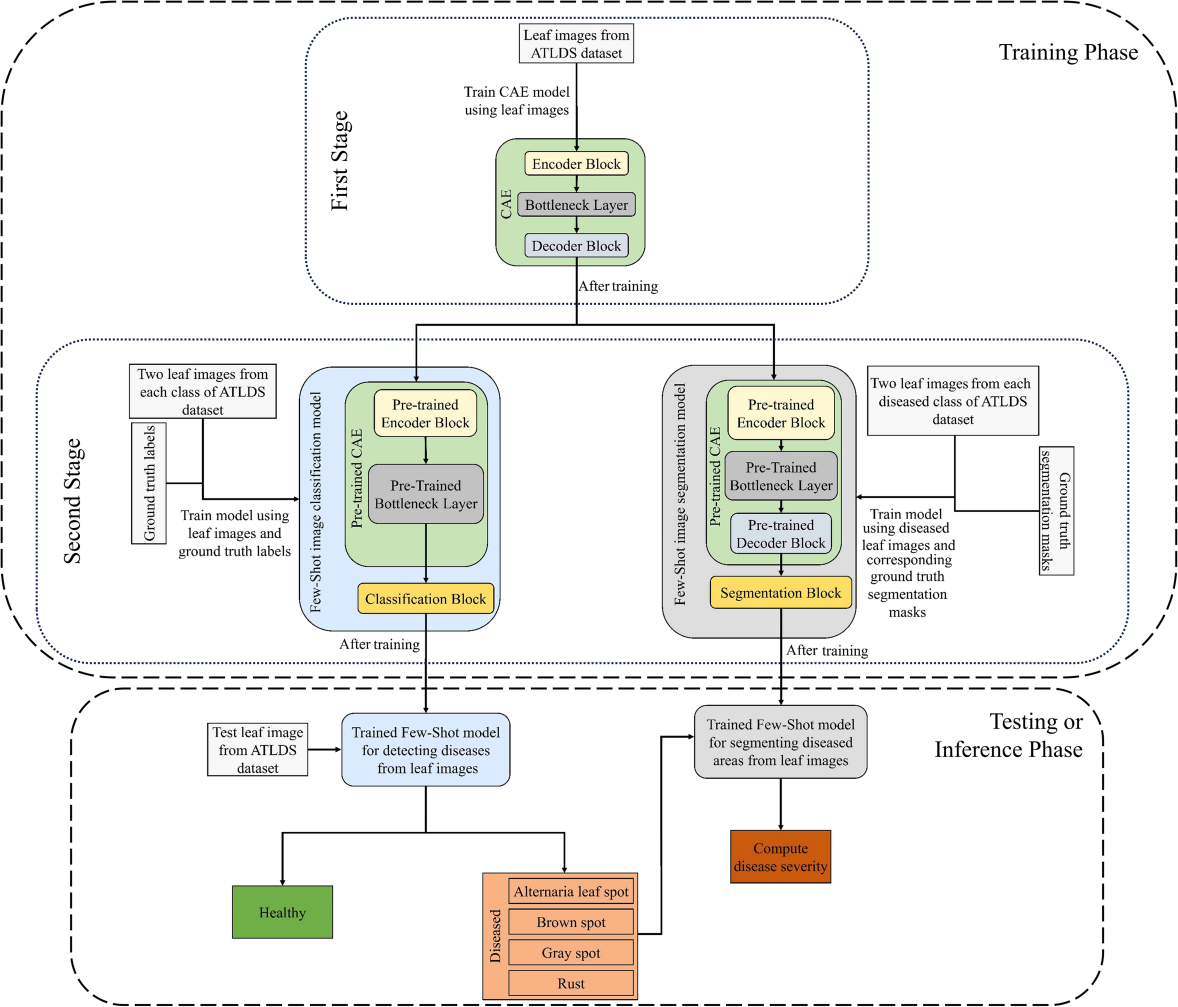
Flow diagram of proposed PDSE-Lite framework’s training and testing phase.

This paper proposes a few-shot and lightweight framework named “PDSE-Lite” based on CAE and FSL for automatic plant disease detection and severity quantification. The PDSE-Lite framework’s flow diagram has been given in **Figure 1**. The motivation to build such type of framework comes from the hypothesis that if a DL model (i.e., CAE) can reconstruct leaf images from original leaf images with minimal information loss, then leveraging its learned knowledge will enable the development of DL models for detecting plant diseases and segmenting disease areas from leaf images using limited training data. Thus, in order to design the PDSE-Lite framework, first, a lightweight CAE model has been designed and trained to reconstruct the leaf images from original leaf images with minimum reconstruction loss. Thereafter, in second stage, a few-shot image classification and segmentation models are built by utilizing the pre-trained layers of the CAE model to detect plant diseases and segment diseased areas from leaf images, respectively. The details of these models have been provided in subsections 3.1, 3.2, and 3.3. After training the few-shot image classification and segmentation models, these models are further utilized in the testing or inference stage to detect plant diseases and estimate the severity of identified diseases by computing the percentage of diseased pixels out of total leaf pixels.

### 3.1 Lightweight Convolutional Autoencoder (CAE)

**Figure 2:**
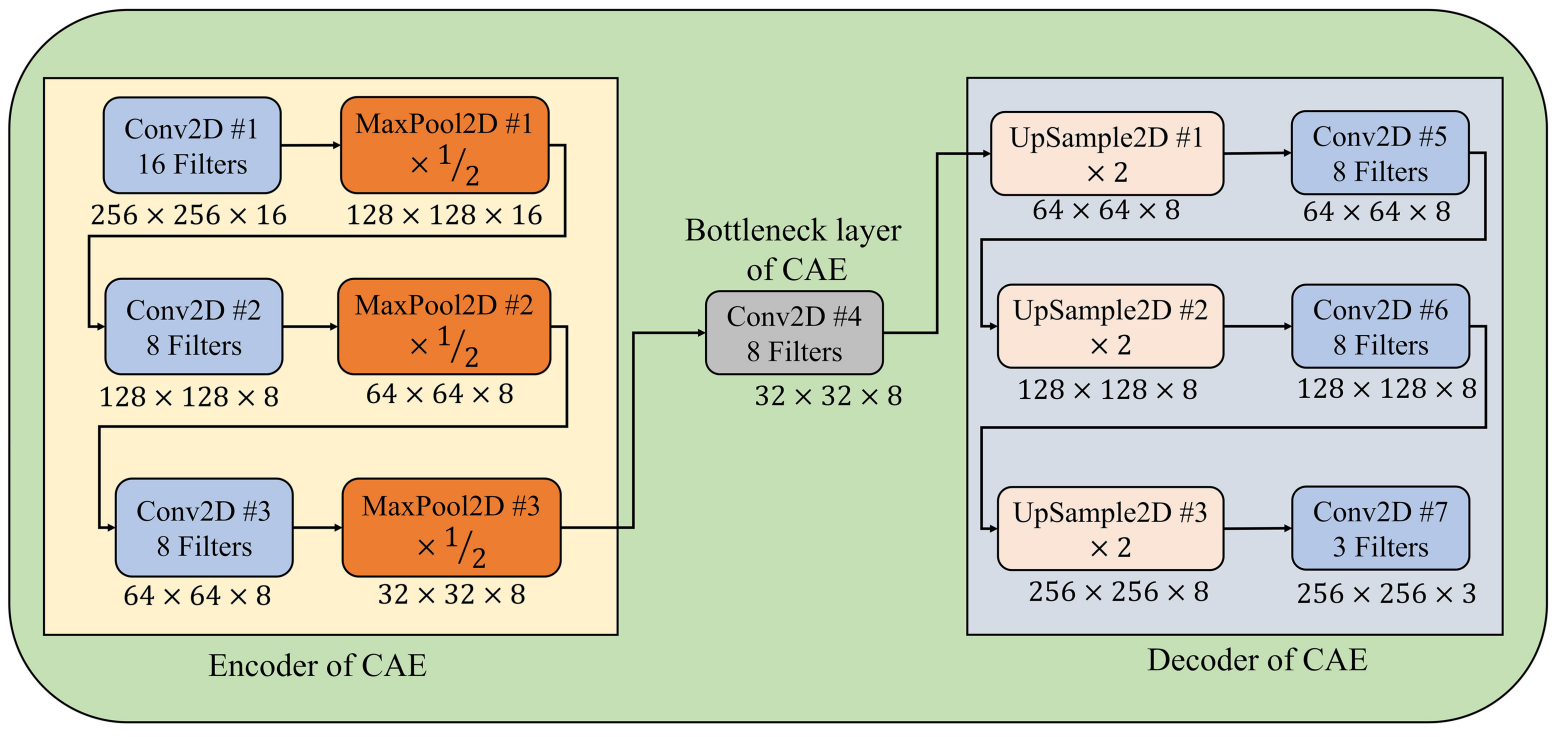
Architectural diagram of the CAE model.


The first stage of proposed PDSE-Lite framework focuses on learning to reconstruct the leaf images from original leaf images with minimum reconstruction loss, and this learning has been done via training a lightweight CAE model. The Convolutional Autoencoder (CAE) is a type of Autoencoder which effectively and efficiently deals with image data as compared to other types of Autoencoders. Like other Autoencoders, CAE also encompasses of one encoder block, bottleneck layer, and decoder block. The encoder block of CAE captures different spatial features of leaf images with the help of multiple convolutional and downsampling (max-pooling) layers and encodes them to a compressed domain representation. This compressed domain representation is stored in the bottleneck layer of CAE, and it comprises of all essential features which are further used by the decoder block of CAE to reconstruct leaf images with minimum reconstruction loss. The decoder block of CAE comprises of same number of layers as of encoder block but in reverse order and upsampling layers are utilized instead of downsampling (max-pooling) layers (Bedi and Gole, 2021a). The CAE model’s encoder block used in this research work comprises of three convolutional layers, each succeeded by a max-pooling layer that decreases the feature map’s spatial dimensionality via factor of two. Similarly, the decoder block of the CAE model also encompasses of three convolutional layers, each preceded by an UpSample layer, which increases the feature map’s spatial dimensionality via factor of two (Bedi and Gole, 2021a). The architectural diagram of CAE model utilized in the PDSE-Lite framework’s first stage has been shown in **Figure 2**, and its implementation details are given in **Supplementary Table 1**. This model has been trained via the Backpropagation algorithm to minimize the Normalized Root Mean Squared Error (NRMSE) reconstruction loss (Feng et al., 2015). The mathematical formula to compute the NRMSE loss (denoted by $Loss_{NRMSE}$ ) between $k^{th}$ input leaf image $I^{k}$ and reconstructed leaf image $R^{k}$ has been given in Equation 1, where N represents total number of leaf images, $Max_{p}$ and $Min_{p}$ represent maximum and minimum values of any pixel present in leaf images (i.e., 255 and 0), respectively.

(1) $$Loss_{NRMSE}=\frac{\sqrt{\frac{1}{N}\sum_{k=1}^{N}{\left(I^{k}-R^{k}\right)}^{2}}}{Max_{p}-Min_{p}}$$

### 3.2 Few-shot image classification model for detecting diseases from leaf images

**Figure 3:**
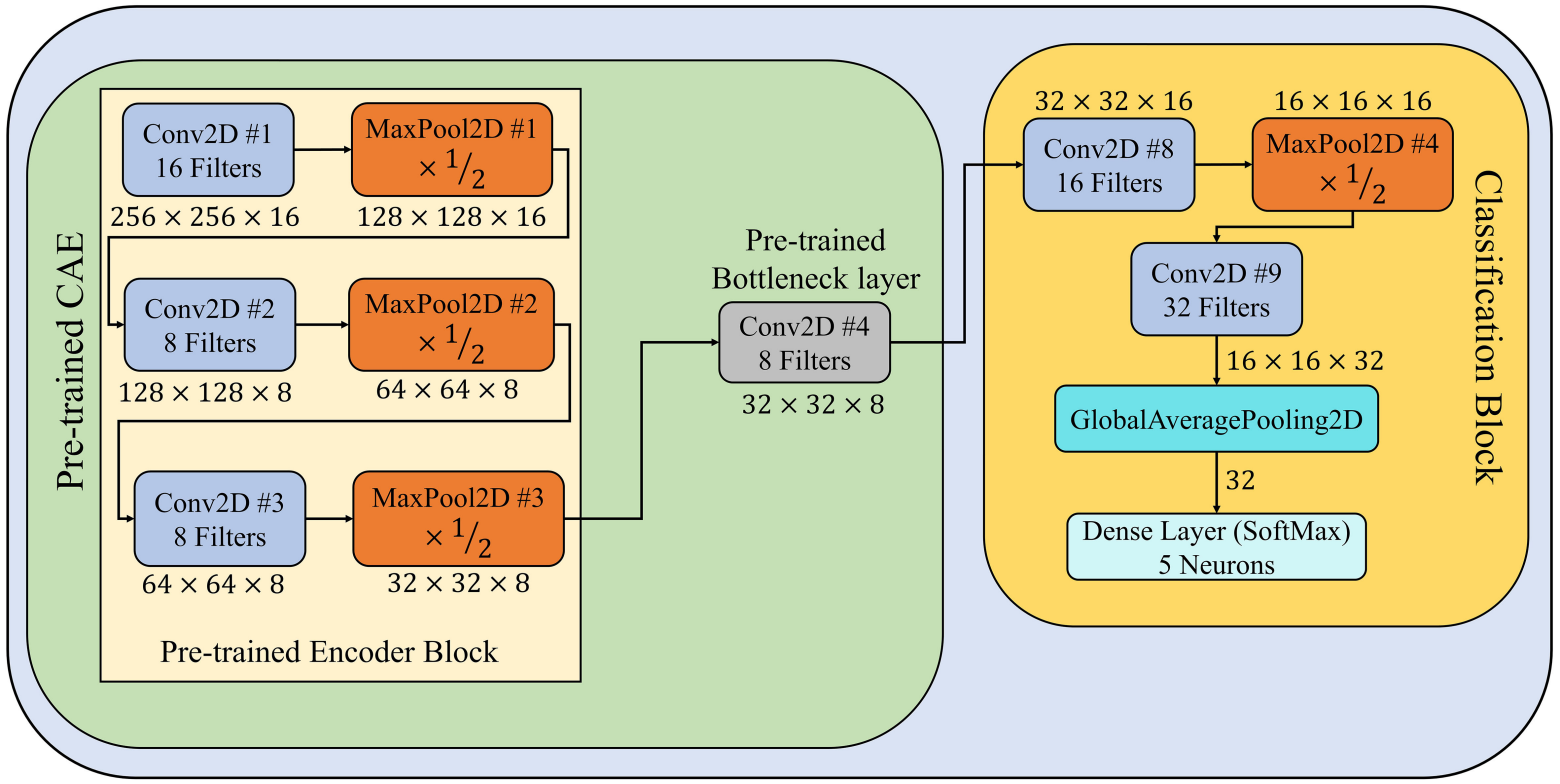
Architectural design of few-shot image classification model used for detecting diseases from leaf images.


During second stage of the PDSE-Lite framework, a few-shot image classification model is designed and developed to identify diseases in plants by using their leaf images. This model encompasses of pretrained encoder block and bottleneck layer of CAE model discussed in subsection 3.1. Furthermore, a classification block is appended ahead of the CAE’s pretrained bottleneck layer in order to fine-tune this model for plant disease recognition. The classification block comprises of two convolutional layers, one max-pooling, global-average-pooling, and dense layers. The architectural design of this model has been shown in **Figure 3**, and its implementation details are given in **Supplementary Table 2**. The training of this model has been done on few training instances using the Backpropagation algorithm, which minimizes the categorical crossentropy loss (denoted by $\;Loss_{CCE}$ ) between predicted labels $Y_{pred}$ and actual labels $Y_{true}$ of leaf images. The mathematical formula of categorical crossentropy loss, i.e., $Loss_{CCE}$ is shown in Equation 2, where N represents number of instances taken into account, $Y_{pred}^{i}$ denotes the predicted label of $i^{th}$ instance, and $Y_{true}^{i}$ represents the actual label of $i^{th}$ instance.

(2) $$Loss_{CCE}=-\frac{1}{N}\sum_{i=1}^{N}Y_{true}^{i}\log\left(Y_{pred}^{i}\right)$$

### 3.3 Few-shot image segmentation model for segmenting disease areas from diseased leaf images

**Figure 4:**
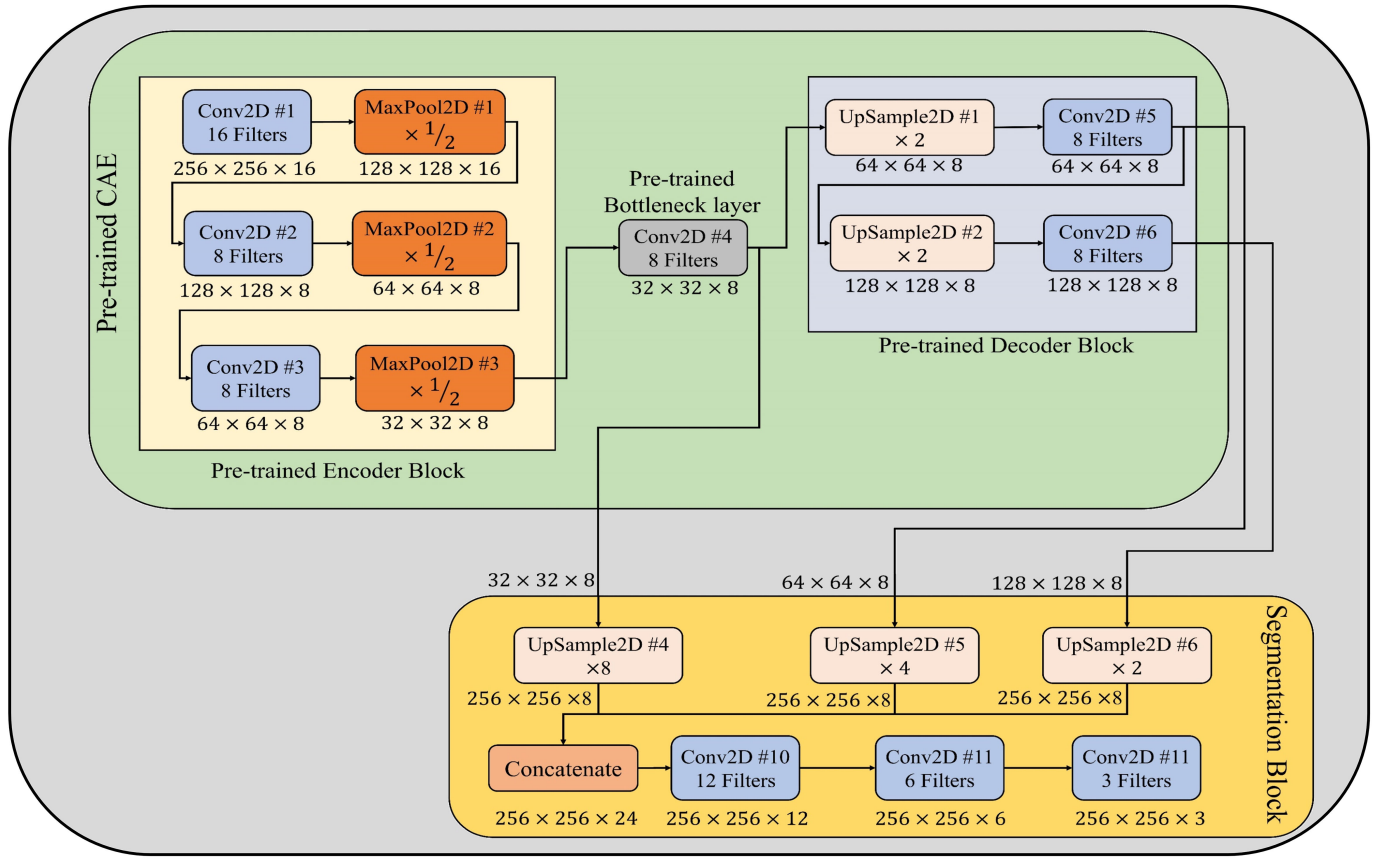
Architectural design of few-shot image segmentation model used to segment diseased areas from leaf images.

In order to estimate the severity of detected plant disease via the few-shot image classification model described in section 3.2, a few-shot image segmentation model has been designed and implemented for segmenting disease areas from leaf images. This model encompasses of pretrained CAE (described in subsection 3.1) and segmentation block. In the segmentation block, first, the output features maps of pretrained bottleneck, Conv2D #5, and Conv2D #6 layers are upsampled by a factor of 8, 4, and 2, respectively. Thereafter, these up-sampled feature maps have been concatenated channel-wise. By this concatenation, all features extracted by different convolutional layers of the CAE model’s decoder block are merged to form a combined feature map. Subsequently, this combined feature map is passed to three stacked convolutional layers having 12, 6, and 3 filters, respectively. The last convolutional layer, which has three filters, acts as an output layer that generates the segmentation mask for a given leaf image. Each pixel of this segmentation mask can have either of three values, i.e., 0 is for the background, 1 is for leaf pixels, and 2 is for diseased pixels. Furthermore, the architectural design of few-shot image segmentation model is depicted in **Figure 4**, and its implementation details have been given in **Supplementary Table 3**.

This few-shot model for segmenting disease areas from leaf images has been trained on few leaf images from diseased classes of the ATLDS dataset, as its output is only needed when the leaf images are classified into any diseased class. This model is also trained using the Backpropagation algorithm, which minimizes the sum of categorical cross-entropy loss ( $SegLoss_{CCE}$ ) and jaccard loss ( $SegLoss_{jaccard}$ ) between predicted and ground truth segmentation masks. The mathematical formulas for $SegLoss_{CCE}$ and $SegLoss_{jaccard}$ are given in Equations 3, 4 correspondingly. In these equations, $Y_{pmask}^{i}$ and $Y_{gmask}^{i}$ represents predicted and ground truth segmentation masks for $i^{th}$ leaf image, respectively. Furthermore, N denotes the number of instances taken into consideration.


(3) $$SegLoss_{CCE}=-\frac{1}{N}\sum_{i=1}^{N}Y_{gmask}^{i}\log\left(Y_{pmask}^{i}\right)$$

(4) $$SegLoss_{jaccard}=1-\frac{1}{N}\sum_{i=1}^{N}\frac{\left|Y_{gmask}^{i}\cap^{\;}Y_{pmask}^{i}\right|}{\left|Y_{gmask}^{i}\cup^{\;}Y_{pmask}^{i}\right|}$$

The flow of plant disease diagnosis and severity quantification through the proposed framework is given in the testing or inference stage of **Figure 1**. It can be observed from this figure that, in order to diagnose disease in a symptomatic leaf image, first, it is passed through the trained few-shot image classification model, which classifies the given leaf image into either healthy or one of diseased classes. If the given leaf image is classified as diseased, then only it is passed to the few-shot image segmentation model, which generates its segmentation mask. This segmentation mask encompasses of three values, i.e., 0 is for background, 1 is for leaf pixels, and 2 is for diseased pixels. After getting the segmentation mask from the few-shot image segmentation model, the disease severity is calculated via computing the percentage of diseased pixels present in the given leaf image out of the total leaf pixels. The formula to compute the disease severity with the help of predicted segmentation mask has been given in Equation 5. In next section, the experimentations performed in this research work have been discussed.


(5) $$Disease\;Severity\;\left(in\;\%\right)=\frac{Total\;number\;of\;diseased\;pixels}{Total\;number\;of\;leaf\;pixels\;}\times 100$$

## 4 Experimental study

The Apple-Tree-Leaf-Disease-Segmentation (ATLDS) dataset is utilized in this research work to test the applicability of PDSE-Lite framework. The description of ATLDS dataset has been given in subsection 4.1, and in subsection 4.2, details of experimentations done in this research work have been provided.

### 4.1 Dataset description

**Figure 5:**
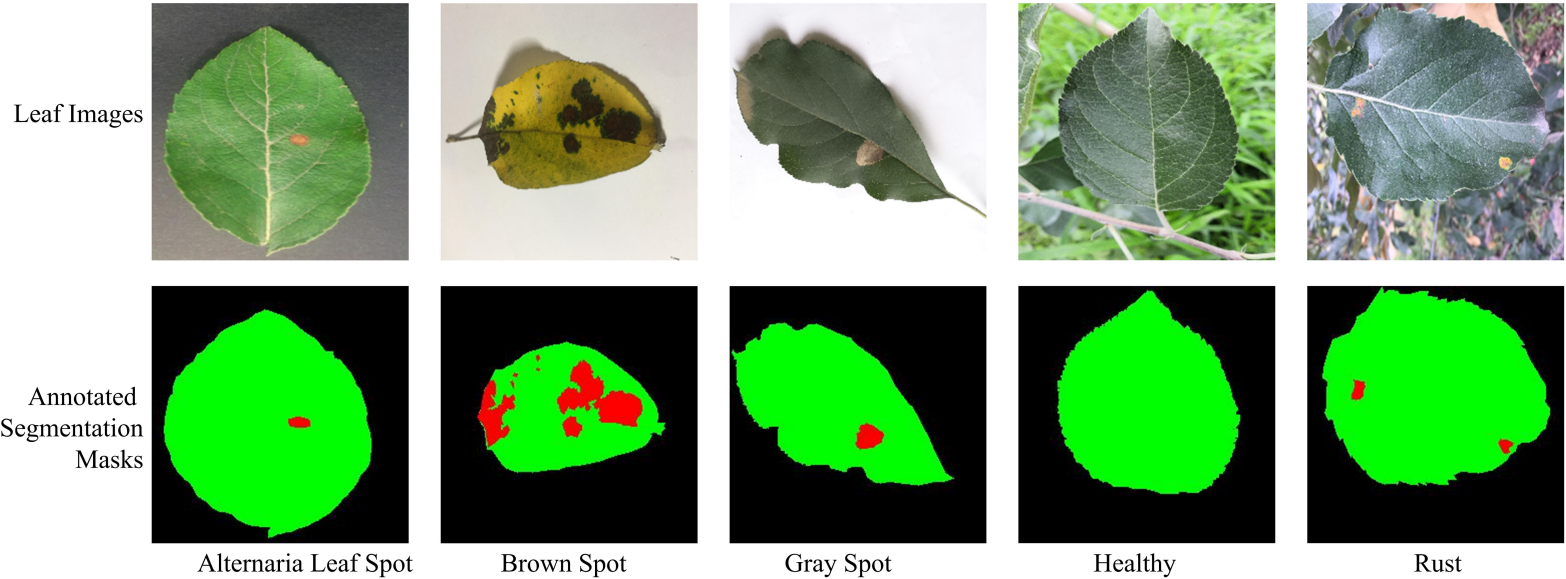
Leaf images representing each class within ATLDS dataset, along with their annotated segmentation masks. The black, green, and red colors in segmentation masks represent the background, leaf, and diseased pixels, respectively.

In this manuscript, the ATLDS dataset (Feng and Chao, 2022) has been employed to test the effectiveness of PDSE-Lite framework in detecting plant diseases with severity estimation. This dataset comprises of healthy and four types of diseased apple tree leaf images, i.e., Alternaria Leaf Spot, Brown Spot, Gray Spot, and Rust. The leaf images of ATLDS dataset were captured under varying degrees of disease, with approximately 51.9% acquired in controlled laboratory settings and 48.1% collected from real cultivation fields. These images were gathered across varied weather conditions and different times of the day. Furthermore, this dataset comprises of annotated segmentation masks corresponding to each leaf image of this dataset. Few leaf images from each class of the ATLDS dataset, along with their annotated segmentation masks, are given in **Figure 5**, and their class-wise distribution is presented in **Table 2**.

**Table 2:** Class-wise distribution of ATLDS dataset.

Class/Type of Leaf image	Alternaria Leaf Spot	Brown Spot	Gray Spot	Healthy	Rust	Total
**Number of Instances**	278	215	395	409	344	**1641**

### 4.2 Experimental setup

This research work involves utilizing the Nvidia DGX Server, which has been equipped with an Intel Xeon CPU, 528 Gigabytes of RAM, and NVidia Tesla V100-SXM2 32 Gigabyte GPU, to conduct experiments. The scripts for the experimentation are written in the Python programming language, although other programming languages can also be used for experimentation. Furthermore, the models of proposed framework are implemented using the Keras library, which is embedded in Tensorflow 2.6.0. The proposed framework has been designed and implemented in two stages. In first stage, a lightweight CAE model has been built to reconstruct leaf images from the original leaf images with minimum reconstruction loss, and subsection 4.2.1 provides the details of the experimentation done to train and test this model. Moreover, in the second stage, a few-shot image classification and segmentation models are developed to identify plant diseases and segment diseased areas from symptomatic leaf images. The details of experimentation done to train and test these models are given in subsections 4.2.2 and 4.2.3.

#### 4.2.1 Experiment 1: training CAE model of the PDSE-Lite framework

In first stage of the PDSE-Lite framework, a lightweight CAE model is designed and developed to reconstruct leaf images from original leaf images with minimal reconstruction loss. In order to train this model, the ATLDS dataset’s leaf images are randomly arranged into training, validation, and testing subsets with 70:15:15 ratio of sizes. The details of leaf images present in training, validation, and testing subsets are given in **Supplementary Table 4**. This model is trained via Adam optimizer to minimize the NRMSE reconstruction loss (defined in Equation 1). During training of the CAE model, the batch size has been kept as 32, and number of epochs are 500. Furthermore, the Rectified Linear Units (ReLU) activation function is applied on every convolutional layer of the CAE model. In order to prevent this model from overfitting, the Earlystopping callback of Keras has been utilized with patience value of 20. The values of these hyperparameters have been obtained through extensive experimentation.

#### 4.2.2 Experiment 2: training few-shot image classification model of the PDSE-Lite framework for plant disease detection

In second stage of the PDSE-Lite framework, a few-shot image classification model has been developed to detect plant diseases through their digital leaf image. To assess this model’s ability toward identifying plant disease with limited training data, it has been trained on k -training-samples per class of ATLDS dataset, where k∈{1,2,…, 5} . Furthermore, $\frac{N_{c}-k}{2}$ and $\frac{N_{c}-k}{2}$ leaf images from different classes of dataset have been utilized for validating and testing the few-shot image classification model, where $N_{c}$ is the number of leaf images present in $c^{th}$ class. This model is trained for 100 epochs with a batch size of k to minimize categorical crossentropy loss (defined in Equation 2) using the Adam optimizer, and the ReLU activation function is utilized in every convolutional layer of the model. Additionally, Early stopping callback of Keras with patience value 10 is applied during model training to prevent it from overfitting. Extensive experimentation has been conducted to determine the values of the aforementioned hyperparameters. This model’s performance is compared with eight different state-of-the-art CNN architectures, i.e., MobileNetV2 (Sandler et al., 2018), InceptionV3 (Szegedy et al., 2016), GoogLeNet (Szegedy et al., 2015), Xception (Chollet, 2017), ResNet-50 (He et al., 2016), NASNetMobile (Zoph et al., 2018), EfficientNetV2B0 (Tan and Le, 2021), and ConvNeXtTiny (Liu et al., 2022).

#### 4.2.3 Experiment 3: training few-shot image segmentation model of the PDSE-Lite framework to segment diseased areas from diseased leaf images

In order to quantify the severity of detected plant disease between 0% to 100%, a few-shot image segmentation model has also been implemented in second stage of the PDSE-Lite framework. This model has been trained on k∈{1,2,…,5} , leaf images from the dataset’s diseased classes, as its output is only needed when a leaf image is classified into one of the diseased class by the few-shot image classification model described in section 3.2. On the other hand, remaining $\frac{N_{c}-k}{2}$ and $\frac{N_{c}-k}{2}$ leaf images from different diseased classes of the ATLDS dataset divided into validation and testing subsets, respectively. Furthermore, this few-shot image segmentation model has also been trained with a batch size of k for 100 epochs to minimize the sum of $SegLoss_{CCE}$ and $SegLoss_{jaccard}$ (defined in Equations 3, 4) via Adam optimizer. Early stopping callback with patience value 10 is applied during model training to stop the model from overfitting. The performance of this few-shot image segmentation model has been compared with U-Net3+ (Huang et al., 2020) and DeepLabV3+ (Chen et al., 2018) image segmentation models using MeanIoU and Dice-Score metrics. The mathematical formulas of MeanIoU and Dice-Score metrics have been given in Equations 6, 7, respectively. In these equations, $Y_{pmask}^{i}$ , and $Y_{gmask}^{i}$ represents predicted and ground truth segmentation masks for $i^{th}$ leaf image, respectively. Additionally, N denotes the number of instances taken into consideration. In this section, the experimental details of the research work are discussed, and in the next section, the experimental results obtained from the experimentation are presented.


(6) $$MeanIoU=\frac{1}{N}\sum_{i=1}^{N}\frac{\left|Y_{gmask}^{i}\cap^{\;}Y_{pmask}^{i}\right|}{\left|Y_{gmask}^{i}\cup^{\;}Y_{pmask}^{i}\right|}$$

(7) $$Dice-Score=\frac{1}{N}\sum_{i=1}^{N}\frac{2\times \left|Y_{gmask}^{i}\cap^{\;}Y_{pmask}^{i}\right|}{\;\;\;\;\left|Y_{gmask}^{i}\right|+\left|Y_{pmask}^{i}\right|}$$

## 5 Experimental results

In this research work, a few-shot and lightweight framework named “PDSE-Lite” has been designed and developed for detecting plant diseases and estimating the severity of identified disease by utilizing digital plant leaf images. During the first stage of the proposed framework’s development, a lightweight CAE model is built, which aims to learn reconstructing leaf images from original leaf images without losing much information. This CAE model’s training, validation, and testing results have been given in subsection 5.1. In the second stage of proposed framework’s development, a few-shot image classification and segmentation models are implemented. The results obtained from the training, validation, and testing phases of these models have been provided in subsections 5.2 and 5.3, respectively. In subsection 5.4, an ablation study to test the significance of pre-trained CAE model has been presented. Moreover, subsection 5.5 provides the statistical analysis of the proposed PDSE-Lite framework.

### 5.1 Results obtained from experiment 1

**Figure 6:**
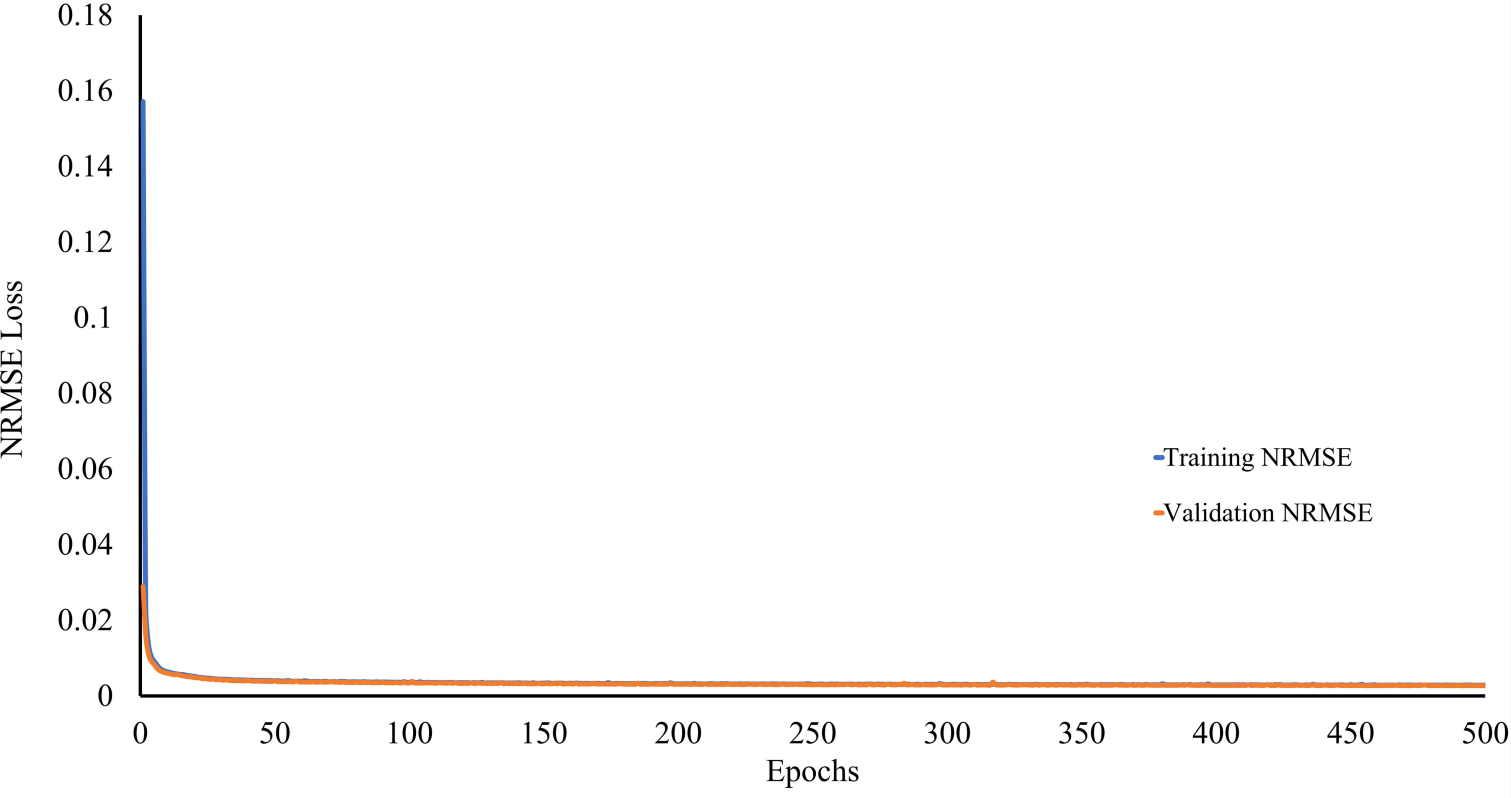
Trend of CAE model’s training and validation NRMSE loss.

**Figure 7:**
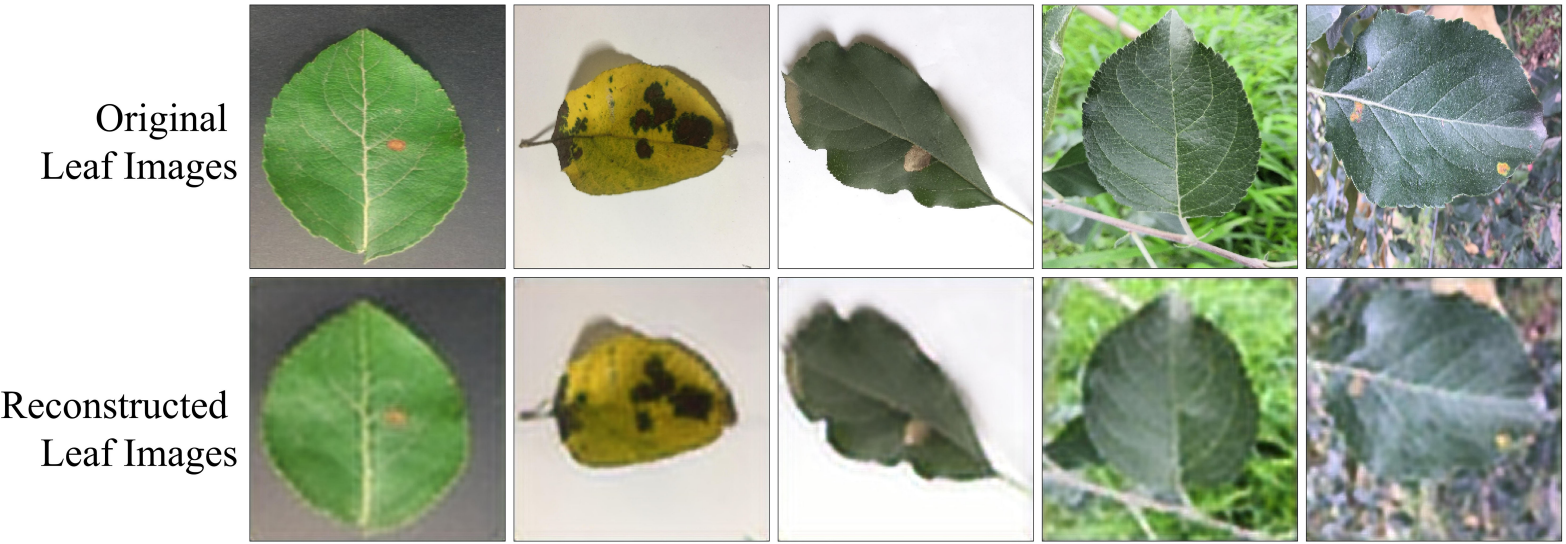
Few leaf images and their reconstructed images from each class of ATLDS dataset using the CAE model of PDSE-Lite framework.

During the first phase of proposed framework’s development, a lightweight CAE model is trained to reconstruct the leaf images from the original leaf images without losing much information. In order to ensure this, the CAE model has been trained to minimize the NRMSE loss (defined in Equation 1) between original and reconstructed leaf images. The trend of CAE model’s training and validation NRMSE loss w.r.t. the number of epochs is shown in **Figure 6**. It can be seen from this figure that both training and validation NRMSE loss of the CAE model have been reduced to 0.002746 and 0.002851 till the end of 500^th^ epoch. Whereas the value of NRMSE loss obtained on the test subset of ATLDS dataset is 0.003. Furthermore, few leaf images and their reconstructed images from each class of the ATLDS dataset using the CAE model have been given in **Figure 7**.

### 5.2 Results obtained from experiment 2

**Figure 8:**
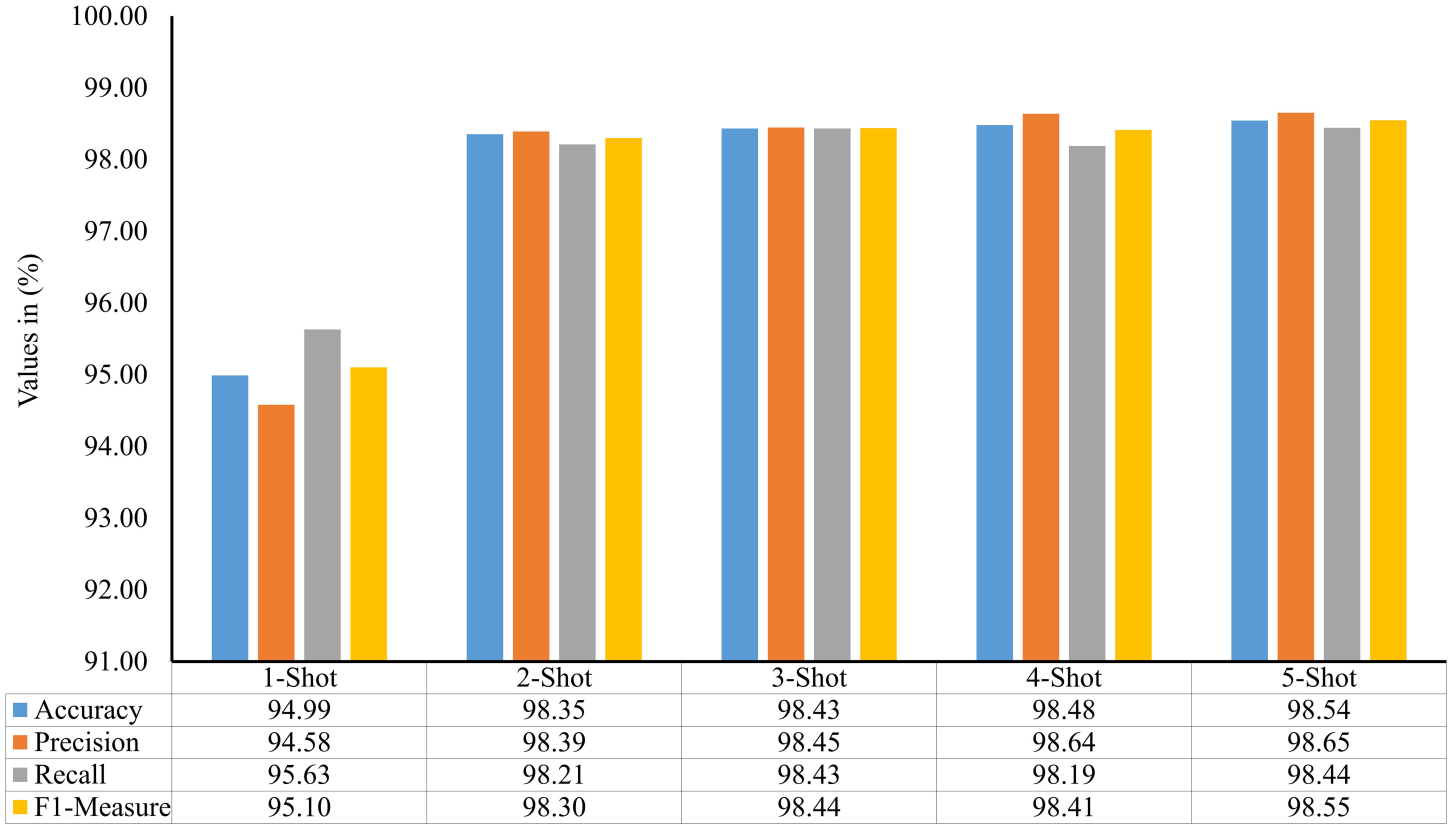
Accuracy, precision, recall, and f1-measure of various few-shot image classification models used to detect plant diseases by visualizing their digital leaf images.

In the second stage of proposed framework’s development, a few-shot image classification model is implemented to diagnose plant diseases through their leaf images. This model’s performance has been evaluated on the ALTDS dataset’s test subset via accuracy, precision, recall, and f1-measure. The comparison of these metrics for different values of k∈{1,2,3,…,5} are given in **Figure 8**. It can be seen by this figure that the 2-shot image classification model has attained 98.35% accuracy and 98.30% f1-measure on the dataset’s test subset. Moreover, the performances of the 3-Shot, 4-Shot, and 5-Shot models are comparable to the 2-Shot model. Therefore, the 2-Shot image classification model has been employed in the PDSE-Lite framework to detect plant diseases, as it requires a minimum number of leaf images for training.

**Figure 9:**
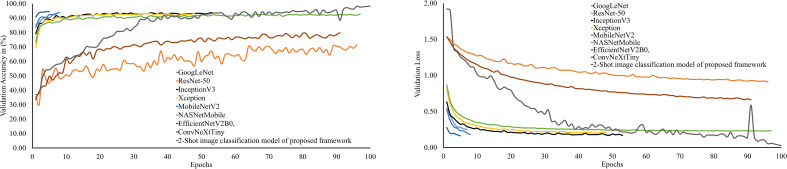
Trend of validation accuracy and validation loss for the 2-Shot image classification model of PDSE-Lite framework and the eight different CNN architectures.

The performance of PDSE-Lite framework’s 2-Shot image classification model on the validation subset of the ATLDS dataset has been compared with eight CNN architectures via validation accuracy and loss. The variation of validation accuracy and loss w.r.t. number of epochs for these models has been depicted in **Figure 9**), respectively. It can be observed from these figures that the 2-Shot image classification model has achieved maximum accuracy and minimum loss, i.e., 98.49% and 0.03, respectively. Furthermore, ResNet-50 achieved minimum accuracy and maximum loss, i.e., 71.40% and 0.91, correspondingly, among other models.

**Figure 10:**
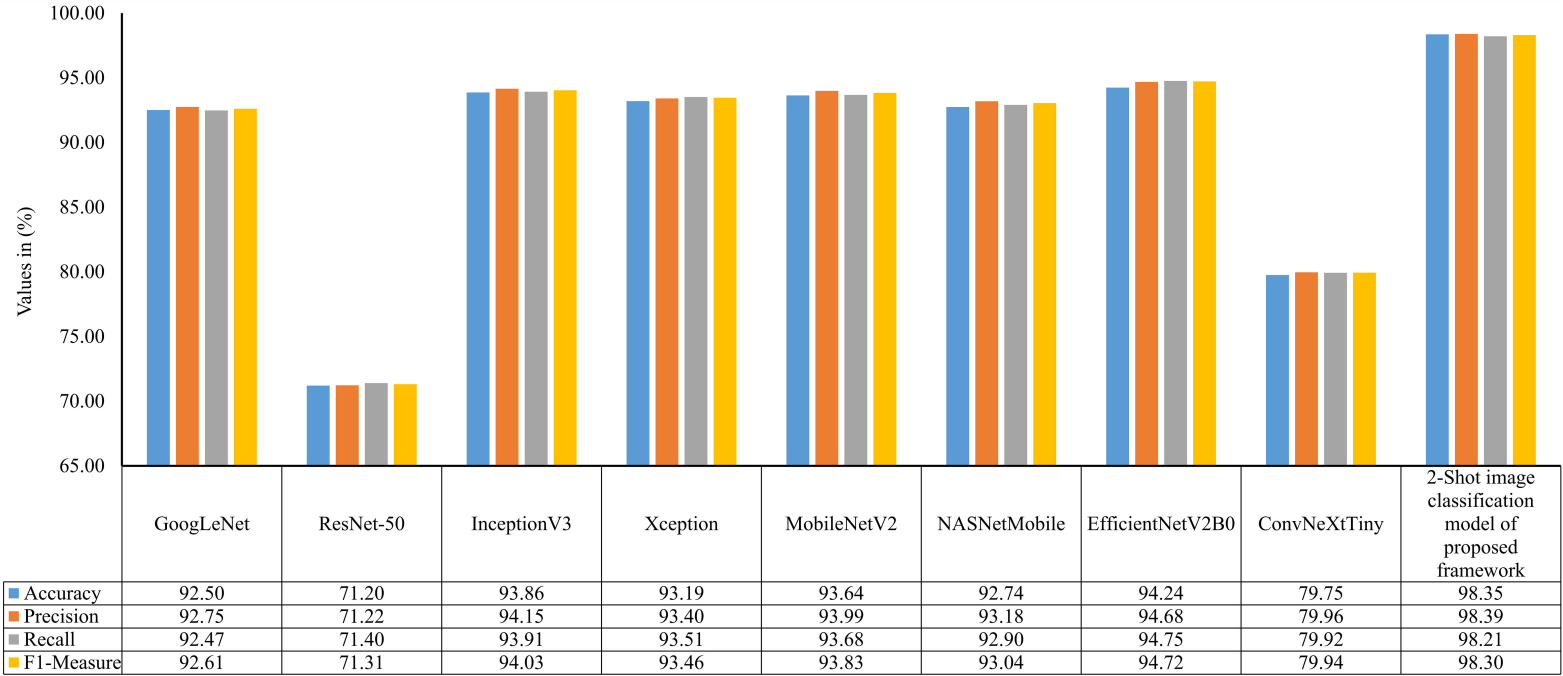
Accuracy, precision, recall, and f1-measure of 2-Shot image classification model of PDSE-Lite framework and eight CNN models on ATLDS dataset’s test subset.

In order to examine the 2-Shot model of PDSE-Lite framework more thoroughly, its performance on the ATLDS dataset’s test subset is compared with eight CNN architectures via accuracy, precision, recall, and f1-measure. The scores of these metrics for the 2-Shot image classification model of PDSE-Lite framework and eight CNN architectures are given in **Figure 10**. It can be perceived from this figure that the 2-Shot image classification model of PDSE-Lite framework outperformed other CNN architectures with 98.35% testing accuracy and 98.30% f1-measure. In addition, GoogLeNet, InceptionV3, Xception, MobileNetV2, NASNetMobile, and EfficientNetV2B0 achieved comparable performance. On the other hand, ResNet-50 and ConvNeXtTiny attained minimum values for the aforementioned metrics.

**Figure 11:**
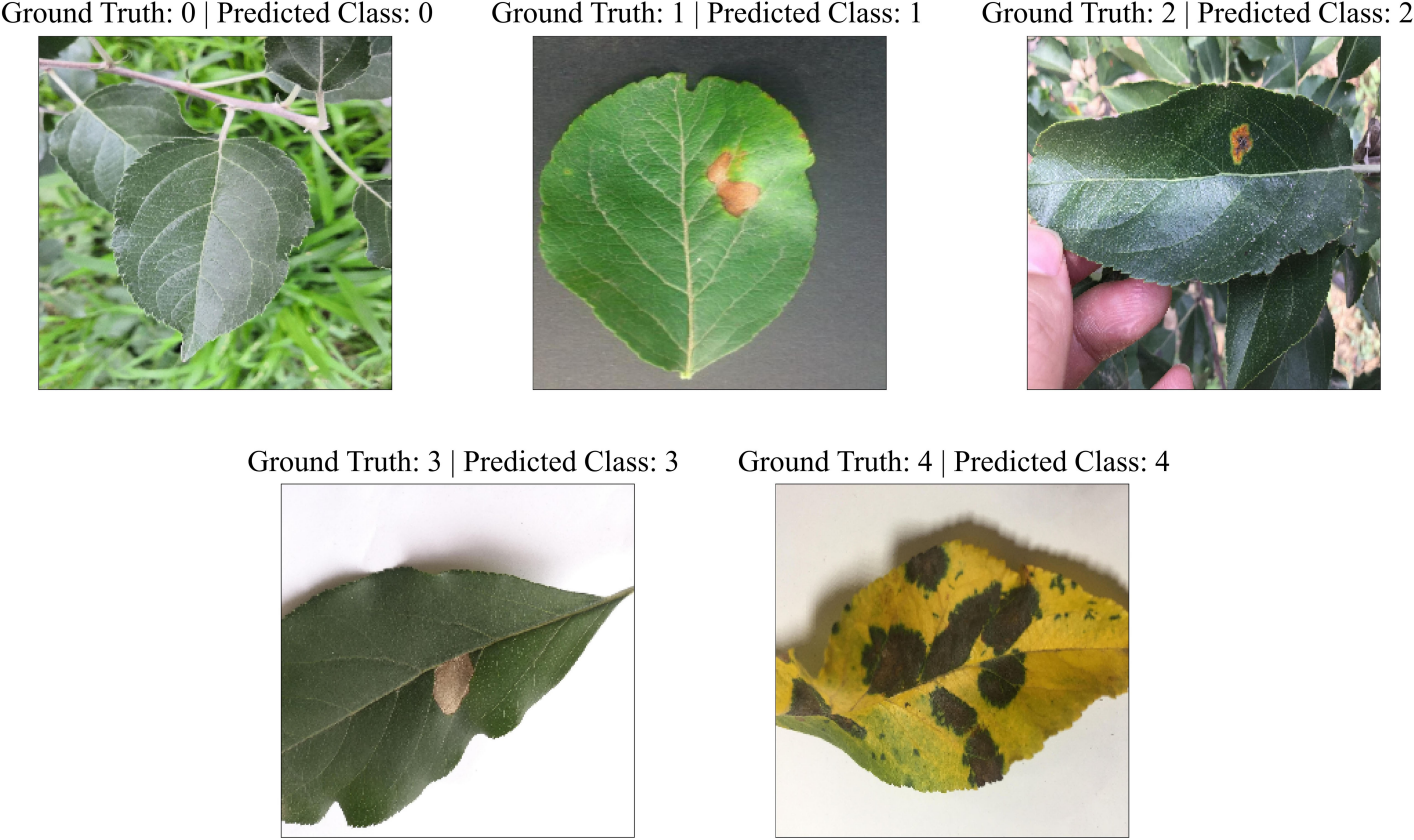
Predictions obtained from the 2-Shot image classification model of PDSE-Lite framework for some sample leaf images from each class of ATLDS dataset, along with their ground truth labels.

In order to examine the lightweight nature of the 2-Shot image classification model, the number of trainable weight parameters employed in this model and eight other state-of-the-art CNN architectures have been compared in **Table 3**. It can be perceived from **Table 3** that the 2-Shot image classification model requires the least trainable weight parameters, i.e., 8749, among other CNN architectures. Furthermore, ResNet-50 and ConvNeXtTiny architectures utilized comparable trainable weight parameters. The predictions obtained from the 2-Shot image classification model for some sample leaf images representing each class within the ATLDS dataset, along with their ground truth labels, have been given in **Figure 11**. It can be perceived from this figure that 2-Shot image classification model correctly identifies the healthy and diseased classes by visualizing the given leaf images.

**Table 3:** Number of trainable weight parameters employed in 2-Shot image classification of PDSE-Lite framework and eight CNN architectures.

Models	Number of trainable weight parameters (approximately)
GoogLeNet	10.32 million
ResNet-50	27.82 million
InceptionV3	21.81 million
Xception	20.87 million
MobileNetV2	2.26 million
NASNetMobile	4.28 million
EfficientNetV2B0	5.93 million
ConvNeXtTiny	27.83 million
**2-Shot image classification model of PDSE-Lite framework**	**8749**

### 5.3 Results obtained from experiment 3

**Figure 12:**
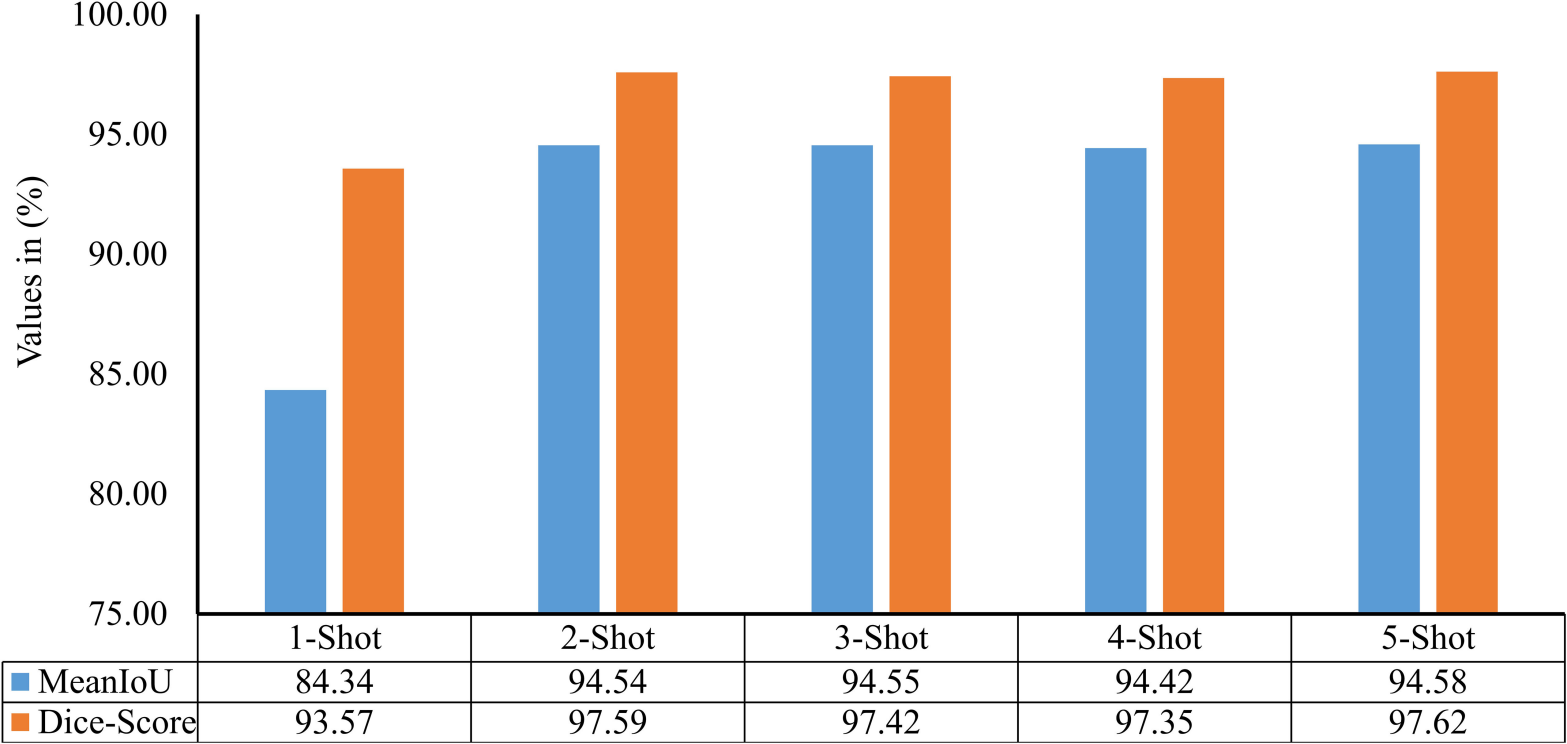
MeanIoU and Dice-Score of various few-shot image segmentation models used to segment plant diseases areas from leaf images.

The model described in section 3.2 only identifies the disease occurrence in a given leaf image. However, it does not quantify the severity of identified disease between 0% to 100%. Thus, a few-shot image segmentation model has also been implemented in second stage of the PDSE-Lite framework’s development. The evaluation of this model has been done on the test subset of the dataset with the help of two widely used evaluation metrics: MeanIoU and Dice-Score, shown in Equations 6, 7. The score of these metrics for different k values is given in **Figure 12**. It can be perceived from this figure that the results obtained for k=2, 3, 4, and 5 are comparable. Thus k=2 , i.e., the 2-Shot image segmentation model has been further used in the proposed framework to segment the diseased from leaf images, as it uses minimum leaf images per class in model training.

**Figure 13:**
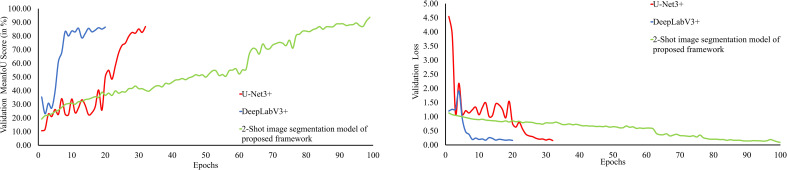
Plot of validation MeanIoU and validation loss for the 2-Shot image segmentation model of PDSE-Lite framework along with U-Net3+ and DeepLabV3+ models.

The performance of the 2-Shot image segmentation model of PDSE-Lite framework has also been evaluated on the validation subset of dataset with the help of validation MeanIoU and validation loss. Furthermore, this model’s performance has been compared with DeepLabV3+ and U-Net3+ image segmentation models. The plot of validation MeanIoU and loss w.r.t. number of epochs for the 2-Shot image segmentation model along with the U-Net3+ and DeepLabV3+ models is given in **Figure 13**, respectively. It can be perceived from these figures that the 2-Shot image segmentation model of PDSE-Lite framework outperformed U-Net3+ and DeepLabV3+ models by achieving the highest validation MeanIoU score, i.e., 94.87% and least validation loss, i.e., 0.09. On the other hand, U-Net3+ and DeepLabV3+ models achieved comparable performances.

**Figure 14:**
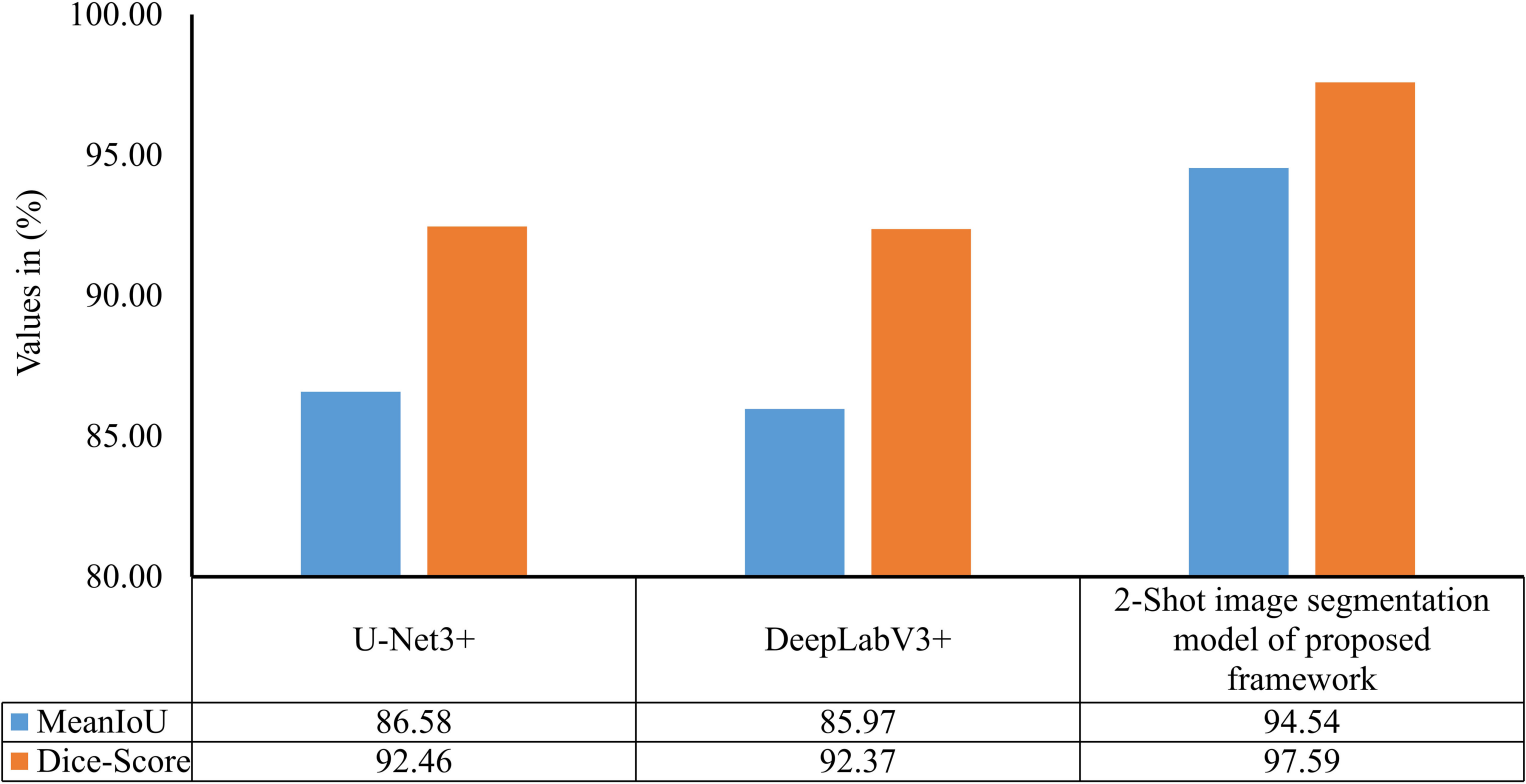
MeanIoU and Dice-Score of 2-Shot image segmentation model of PDSE-Lite framework, U-Net3+, and DeepLabV3+ models.

**Figure 15:**
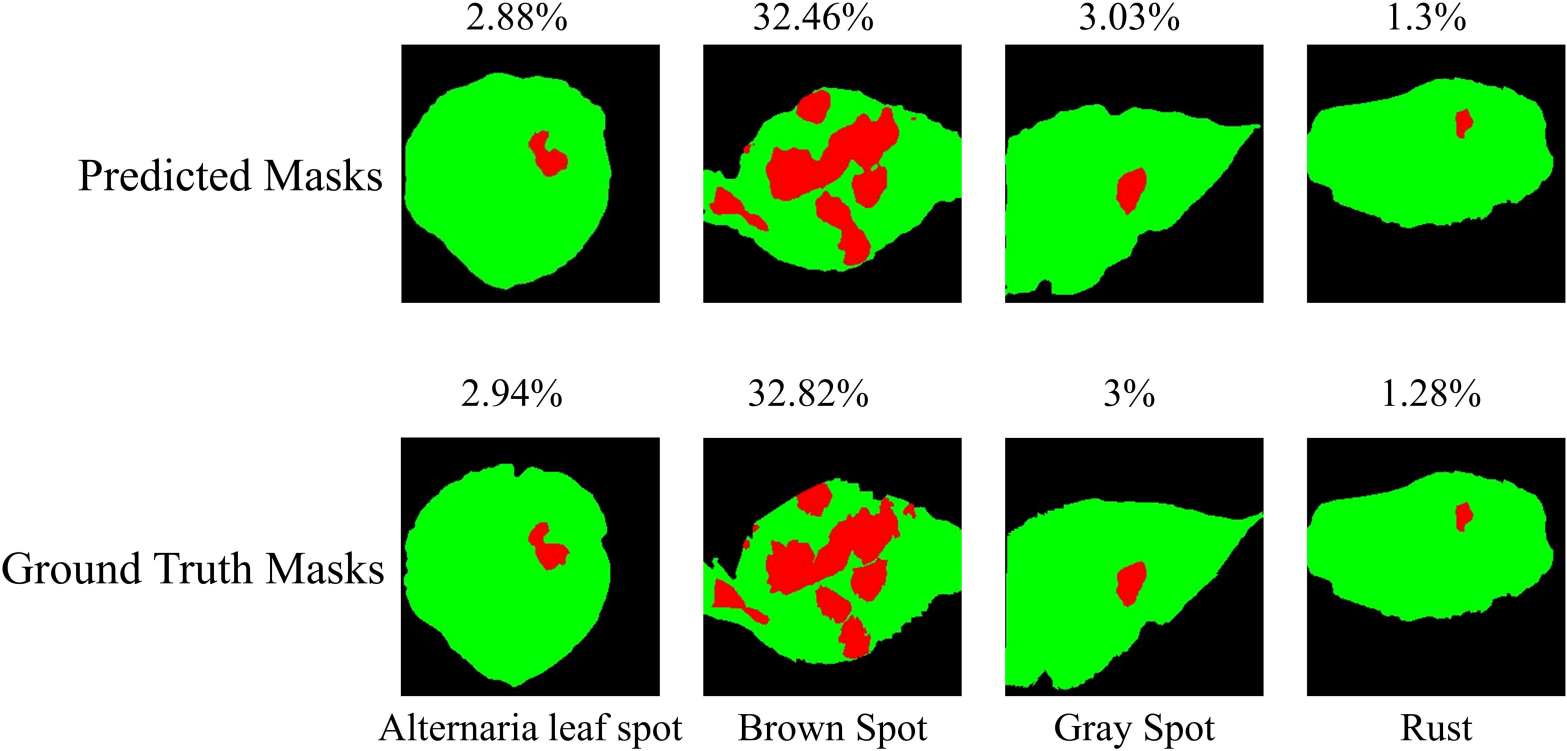
Predicted segmentation masks for some sample leaf images from each diseased class of dataset along with ground truth segmentation masks. The severity percentage obtained from predicted and ground truth segmentation masks have been written above the segmentation masks.

The performance of the 2-Shot image segmentation model has also been compared with the U-Net3+ and DeepLabV3+ model on the test subset of ATLDS dataset using MeanIoU and Dice-Score. This comparison is shown via a bar graph in **Figure 14**. It can be perceived from **Figure 14** that the 2-Shot image segmentation model outperformed the DeepLabV3+ and UNet3+ models on the test subset also by attaining 94.54% and 97.59% MeanIoU score and Dice-Score, respectively. In order to measure the lightweight nature of 2-Shot image segmentation model, its number of trainable weight parameters is compared in **Table 4** with the trainable weight parameters used by U-Net3+ and DeepLabV3+ models. It can be observed from **Table 4** that the 2-Shot image segmentation model uses significantly fewer trainable weight parameters, i.e., 7223. The predicted segmentation masks for some sample leaf images from each diseased class of dataset and ground truth segmentation masks have been given in **Figure 15**. Furthermore, the severity percentages obtained from predicted and ground truth segmentation masks have been computed and written above the segmentation masks. It can be seen from **Figure 15** that predicted and ground truth masks are looking very similar to each other. In addition, the severity percentages computed for these segmentation masks are also comparable, which confirms the effectiveness of the proposed framework in identifying and quantifying plant diseases in the real world.

**Table 4:** Number of trainable weight parameters used by 2-Shot image segmentation model, U-Net3+, and DeepLabV3+ models.

Models	Number of trainable weight parameters (approximately)
U-Net3+	26.99 million
DeepLabV3+	11.85 million
**2-Shot image segmentation model of PDSE-Lite framework**	**7223**

### 5.4 Ablation study to test the significance of pretrained CAE model

In order to test the significance of pretrained CAE model in the 2-Shot image classification and 2-Shot image segmentation models of PDSE-Lite framework, these models are also trained without utilizing the pre-trained CAE model. The results obtained from this experiment on test subset of ATLDS dataset have been tabulated in **Table 5** along with the results of 2-Shot image classification and 2-Shot image segmentation models of PDSE-Lite framework in which the pre-trained CAE model is employed.

**Table 5:** Results obtained with and without utilizing the pre-trained CAE model in the 2-Shot image classification and 2-Shot image segmentation models of PDSE-Lite framework.

Models of PDSE-Lite framework	Without pre-trained CAE model		With pre-trained CAE model	
**2-Shot image classification model**	Accuracy (%)	72.58	Accuracy (%)	98.35
	Precision (%)	74.64	Precision (%)	98.39
	Recall (%)	71.76	Recall (%)	98.21
	F1-Measure (%)	73.17	F1-Measure (%)	98.30
**2-Shot image segmentation model**	MeanIoU (%)	68.99	MeanIoU (%)	94.54
	Dice-Score (%)	72.54	Dice-Score (%)	97.59

### 5.5 Statistical analysis of PDSE-Lite framework

The applicability of PDSE-Lite framework in estimating plant disease severity has also been tested using statistical hypothesis testing via the Student t-test on the severity values obtained for ground truth and predicted segmentation masks. The t-test has been utilized to test the null hypothesis, which states that the severity values calculated by the PDSE-Lite framework are very similar to the severity values obtained by ground truth segmentation masks. The paired t-test with two samples for means assuming unequal variance is employed in this research work to test the null and alternate hypothesis given in Equations 8, 9, respectively.


(8) $$H_{0}≔\mu_{gt}-\mu_{pred}\neq 0$$

(9) $$H_{1}≔\mu_{gt}-\mu_{pred}=0$$

where, $\mu_{gt}:\;$ mean of disease severity values obtained from ground truth segmentation masks $\mu_{pred}:$ mean of disease severity values obtained from predicted segmentation masks.

During experimentation, the probability p value is computed for the t-test at α=0.01 , i.e., if the obtained p value is lesser than 0.01, then the null hypothesis ( $H_{0}$ ) is rejected, and the alternate hypothesis is accepted with 99% confidence. After analyzing the experimental results of the t-test, p value for the t-test is obtained as 0.008, which is lesser than 0.01. Thereby, the null hypothesis is rejected, and the alternate hypothesis is accepted with 99% confidence. Hence, this showcases the applicability of PDSE-Lite framework in precisely estimating plant disease severity. The next section of the paper discusses the results presented in this section.

## 6 Discussion

Plant disease detection with severity estimation is still a prominent challenge in agricultural research. The majority of research works present in the literature utilized large amount of manually annotated plant leaf images to train an ML or DL model for plant disease severity estimation (Wspanialy and Moussa, 2020; Wang et al., 2021a; Ji and Wu, 2022). However, annotating large amount of leaf images is laborious and time-consuming. Therefore, in this research work, a few-shot and lightweight framework named “PDSE-Lite” based on CAE and FSL has been proposed to reduce the reliance on large-scale manually labeled datasets and offer a promising solution for early-stage plant disease detection with severity estimation. The proposed framework is designed and developed in two stages. In the first stage of proposed framework’s development, a lightweight CAE model (Bedi and Gole, 2021a) is built and trained to efficiently reconstruct leaf images from original leaf images with minimal reconstruction loss. The layers of pretrained CAE model are then utilized to build a few-shot image classification and segmentation models. These models are subsequently trained with a limited number of leaf images to accurately detect plant diseases and precisely segment the diseased regions from leaf images for severity estimation. Thereafter, the disease severity is calculated by computing the percentage of diseased leaf pixels obtained through segmentation out of the total leaf pixels.

To assess the PDSE-Lite framework’s applicability, it is trained and tested on ATLDS dataset comprising of diseased and healthy leaf images of apple trees along with their segmentation masks. Experimental results revealed that the CAE model used in the first phase of proposed framework’s development can reconstruct the given leaf images from original leaf images without losing much information, as very low value of NRMSE loss, i.e., 0.003, is obtained during experimentation (discussed in section 5.1). In this research work, five variants of the few-shot image classification and segmentation models have been implemented and trained on k∈{1,2,3,…,5} leaf images representing each class within ATLDS dataset. Further, these variants are referred as 1-Shot, 2-Shot, 3-Shot, 4-Shot, and 5-Shot image classification and segmentation models, respectively. It can be observed from **Figures 8**, **12** that 2-Shot, 3-Shot, 4-Shot, and 5-Shot variants of few-shot image classification and segmentation models have achieved comparable performances. Thus, out of these variants, the 2-Shot variant of image classification and segmentation models is exploited in the proposed PDSE-Lite framework to identify plant diseases from leaf images and segment diseased areas from leaf images, as it uses the minimum leaf images for training. The performance of the 2-Shot image classification model of PDSE-Lite framework is compared with eight CNN architectures, and it is found that the 2-Shot image classification model outperformed other CNN architectures by achieving 98.35% testing accuracy. Additionally, the performance of 2-Shot image segmentation model of PDSE-Lite framework has been compared with DeepLabV3+ and UNet3+ image segmentation models. After comparison, it is found that the 2-Shot image segmentation model achieved 97.59% Dice-Score and 94.54% MeanIoU. The proposed framework’s applicability has also been verified with the help of statistical hypothesis testing via applying the Student t-test on the severity values obtained from predicted and ground truth segmentation masks. After analyzing the Student t-test results, it is found that the PDSE-Lite framework can accurately estimate the severity of plant diseases with 99% confidence interval.

The proposed PDSE-Lite framework is compared in **Table 6** with existing state-of-the-art research works available in the literature. It can be seen from this table that despite of using minimum trainable weight parameters and limited number of training samples, the proposed PDSE-Lite framework has achieved state-of-the-art performance in plant disease detection with severity estimation. Hence, it can be concluded that the proposed PDSE-Lite framework has several advantages over existing state-of-the-art research works present in the literature. First advantage of the PDSE-Lite framework lies in its ability to significantly reduce the reliance on large-scale manually annotated datasets, thereby minimizing the human efforts required to create such datasets. Moreover, the lightweight nature of the PDSE-Lite framework makes it suitable to be deployed on low-powered edge devices for on-site plant disease monitoring and timely intervention, aiding farmers in decision-making and crop management. In this research work, the applicability of proposed framework has been evaluated only on the ATLDS dataset. Nevertheless, future research works would involve training on other plant disease detection and severity estimation datasets having broader range of leaf images of various plants suffering from different diseases. Additionally, in the future, the proposed framework can also be deployed on various IoT devices like Unmanned Aerial Vehicles (UAVs) to facilitate real-time plant disease monitoring in agricultural fields.

**Table 6:** Comparison of proposed PDSE-Lite framework with state-of-the-art research works present in the literature.

Research Work	Technique used	Crop	Performance on test subset of the dataset	Few-Shot (✔/✘)	Disease Detection (✔/✘)	Severity Estimation (✔/✘)	Number of trainable parameters used
Chao et al. (2020)	Xception DenseNet (XDNet)	Apple	98.82% accuracy	✘	✔	✘	10.16 million
**PDSE-Lite framework**	**CAE and FSL**	**Apple**	**98.35% accuracy in plant disease detection**	**✔**	**✔**	**✔**	**8749 for few-shot image classification model**
			**97.59% Dice-Score and 94.54% MeanIoU in segmenting diseased areas from leaf images**				**7223 for few-shot image segmentation model**

## 7 Conclusion

Plant disease identification with severity estimation is still a prominent research challenge in front of agricultural scientists, as it has the potential to maximize the crop yield, which further increases the farmer’s profit. In the literature, the majority of research works focused only on plant disease detection. However, a limited number of studies are available on plant disease severity estimation, and all of these research works have used large amount of manually annotated plant leaf images to train their models. Furthermore, creating such dataset is quite a cumbersome and time-consuming task. Hence, in this research work, a few-shot and lightweight framework named “PDSE-Lite” was proposed to recognize plant diseases and estimate the severity of identified disease between 0% to 100%. The PDSE-Lite framework was designed and developed in two stages with the help of CAE and FSL. In the first stage, a lightweight CAE model was used to reconstruct the leaf images from the original leaf images with minimum loss of information. In the second phase of proposed framework’s implementation, a few-shot image classification and segmentation models were developed to accurately identify plant diseases and precisely segment the diseased areas from given leaf images, respectively. The applicability of proposed PDSE-Lite framework was verified on a publicly available ATLDS dataset comprising apple tree leaf images and their annotated segmentation masks. The proposed framework outperformed various state-of-the-art techniques present in the literature by identifying and segmenting diseased areas from apple leaf images with 98.35% accuracy and 97.59% Dice-Score, respectively. Furthermore, the PDSE-Lite framework requires only two images per class of the ATLDS dataset for training, thus significantly reducing the human efforts required to annotate leaf images. To showcase the lightweight nature of the PDSE-Lite framework, the trainable weight parameters utilized by few-shot image classification and segmentation models of the proposed framework were compared with existing state-of-the-art techniques. After analyzing the results of trainable parameter comparison, it was found that the models of the proposed framework require minimum trainable weight parameters, i.e., 8749 and 7223 for image classification and segmentation models, respectively. The applicability of the proposed framework was further verified through statistical hypothesis testing, which employs the Student t-test on severity values extracted from predicted and ground truth segmentation masks. Upon analyzing the results of the Student t-test, it was determined that the PDSE-Lite framework accurately estimated the severity of plant diseases with a 99% confidence interval. In conclusion, the proposed framework effectively addressed the challenge of early-stage plant disease diagnosis and severity estimation without extensive manual data annotation.

In this study, the proposed framework’s effectiveness was only evaluated on the ATLDS dataset. However, in forthcoming research works, it can be trained on different plant disease detection and severity estimation datasets, which comprise of a wider range of leaf images of various plants suffering from different diseases. Furthermore, the future work of this research also includes the deployment of the PDSE-Lite framework on different IoT devices, such as Unmanned Aerial Vehicles (UAVs), to enable real-time monitoring of plant diseases in agricultural fields.

## Data availability statement

Publicly available datasets were analyzed in this study. This data can be found here: https://www.scidb.cn/en/detail?dataSetId=0e1f57004db842f99668d82183afd578.

## Author contributions

PB: Conceptualization, Formal analysis, Methodology, Project administration, Supervision, Writing – review & editing. PG: Conceptualization, Methodology, Software, Visualization, Writing – original draft. SM: Formal analysis, Resources, Writing – review & editing.

## References

[B1] Abbas A. Jain S. Gour M. Vankudothu S. (2021). Tomato plant disease detection using transfer learning with C-GAN synthetic images. Comput. Electron Agric. 187, 106279. doi: 10.1016/j.compag.2021.106279

[B2] Argüeso D. Picon A. Irusta U. Medela A. San-Emeterio M. G. Bereciartua A.. (2020). Few-Shot Learning approach for plant disease classification using images taken in the field. Comput. Electron Agric. 175, 105542. doi: 10.1016/j.compag.2020.105542

[B3] Atila Ü. Uçar M. Akyol K. Uçar E. (2021). Plant leaf disease classification using EfficientNet deep learning model. Ecol. Inform 61, 101182. doi: 10.1016/j.ecoinf.2020.101182

[B4] Barbedo J. G. A. (2014). An automatic method to detect and measure leaf disease symptoms using digital image processing. Plant Dis. 98, 1709–1716. doi: 10.1094/PDIS-03-14-0290-RE 30703885

[B5] Bedi P. Gole P. (2021a). Plant disease detection using hybrid model based on convolutional autoencoder and convolutional neural network. Artif. Intell. Agric. 5, 90–101. doi: 10.1016/j.aiia.2021.05.002

[B6] Bedi P. Gole P. (2021b). “PlantGhostNet: an efficient novel convolutional neural network model to identify plant diseases automatically,” in 2021 9th International Conference on Reliability, Infocom Technologies and Optimization (Trends and Future Directions) (ICRITO), Noida, India. (Noida, India: IEEE), 1–6, IEEE. doi: 10.1109/ICRITO51393.2021.9596543

[B7] Bedi P. Gole P. Agarwal S. K. (2021). “18 Using deep learning for image-based plant disease detection,” in Internet of Things and Machine Learning in Agriculture (Germany: De Gruyter), 369–402. doi: 10.1515/9783110691276-018

[B8] Biswas S. Jagyasi B. Singh B. P. Lal M. (2014). “Severity identification of Potato Late Blight disease from crop images captured under uncontrolled environment,” in 2014 IEEE Canada International Humanitarian Technology Conference - (IHTC), Montreal, QC, Canada. (Montreal, QC, Canada: IEEE), 1–5, IEEE. doi: 10.1109/IHTC.2014.7147519

[B9] Bock C. H. Poole G. H. Parker P. E. Gottwald T. R. (2010). Plant disease severity estimated visually, by digital photography and image analysis, and by hyperspectral imaging. CRC Crit. Rev. Plant Sci. 29, 59–107. doi: 10.1080/07352681003617285

[B10] Chao X. Sun G. Zhao H. Li M. He D. (2020). Identification of apple tree leaf diseases based on deep learning models. Symmetry (Basel) 12, 1065. doi: 10.3390/sym12071065

[B11] Chen L.-C. Zhu Y. Papandreou G. Schroff F. Adam H. (2018). “Encoder-decoder with atrous separable convolution for semantic image segmentation,” in European Conference on Computer Vision (ECCV) 2018, Munich, Germany. 833–851, (Munich, Germany: Springer). doi: 10.1007/978-3-030-01234-2_49

[B12] Chen S. Zhang K. Zhao Y. Sun Y. Ban W. Chen Y.. (2021). An approach for rice bacterial leaf streak disease segmentation and disease severity estimation. Agriculture 11, 420. doi: 10.3390/agriculture11050420

[B13] Chohan M. Khan A. Katper S. Mahar M. (2020). Plant disease detection using deep learning. Int. J. Recent Technol. Eng. 9, 909–914. doi: 10.35940/ijrte.A2139.059120

[B14] Chollet F. (2017). “Xception: deep learning with depthwise separable convolutions,” in 2017 IEEE Conference on Computer Vision and Pattern Recognition (CVPR), (Honolulu, HI, USA: IEEE)., 1800–1807. doi: 10.1109/CVPR.2017.195

[B15] Chowdhury M. E. H. Rahman T. Khandakar A. Ayari M. A. Khan A. U. Khan M. S.. (2021). Automatic and reliable leaf disease detection using deep learning techniques. AgriEngineering 3, 294–312. doi: 10.3390/agriengineering3020020

[B16] Dhiman P. Kukreja V. Manoharan P. Kaur A. Kamruzzaman M. M. Dhaou I.B.. (2022). A novel deep learning model for detection of severity level of the disease in citrus fruits. Electron. (Basel) 11, 495. doi: 10.3390/electronics11030495

[B17] Dhingra G. Kumar V. Joshi H. D. (2018). Study of digital image processing techniques for leaf disease detection and classification. Multimed Tools Appl. 77, 19951–20000. doi: 10.1007/s11042-017-5445-8

[B18] Divyanth L. G. Ahmad A. Saraswat D. (2023). A two-stage deep-learning based segmentation model for crop disease quantification based on corn field imagery. Smart Agric. Technol. 3, 100108. doi: 10.1016/J.ATECH.2022.100108

[B19] Feng D. Feng M. Ozer E. Fukuda Y. (2015). A vision-based sensor for noncontact structural displacement measurement. Sensors 15, 16557–16575. doi: 10.3390/s150716557 26184197 PMC4541893

[B20] Feng J. Chao X. (2022). Apple Tree Leaf Disease Segmentation Dataset. (Beijing, China: Science Data Bank). doi: 10.11922/sciencedb.01627

[B21] Firdous S. Akbar S. Hassan S. A. Khalid A. Gull S. (2023). “Deep convolutional neural network-based framework for apple leaves disease detection,” in 2023 4th International Conference on Advancements in Computational Sciences (ICACS), Lahore, Pakistan: IEEE. 1–6, IEEE. doi: 10.1109/ICACS55311.2023.10089774

[B22] Garg S. Singh P. (2023). An aggregated loss function based lightweight few shot model for plant leaf disease classification. Multimed Tools Appl. 82, 23797–23815. doi: 10.1007/S11042-023-14372-7/TABLES/13

[B23] Gole P. Bedi P. Marwaha S. Haque M. Deb C. K. (2023). TrIncNet: a lightweight vision transformer network for identification of plant diseases. Front. Plant Sci. 14. doi: 10.3389/fpls.2023.1221557 PMC10414585 37575937

[B24] Haque M. Marwaha S. Arora A. Deb C. K. Misra T. Nigam S.. (2022a). A lightweight convolutional neural network for recognition of severity stages of maydis leaf blight disease of maize. Front. Plant Sci. 13. doi: 10.3389/FPLS.2022.1077568/BIBTEX PMC9833299 36643296

[B25] Haque M. Marwaha S. Deb C. K. Nigam S. Arora A. Hooda K. S.. (2022b). Deep learning-based approach for identification of diseases of maize crop. Sci. Rep. 12, 6334. doi: 10.1038/s41598-022-10140-z 35428845 PMC9012772

[B26] He K. Zhang X. Ren S. Sun J. (2016). “Deep residual learning for image recognition,” in 2016 IEEE Conference on Computer Vision and Pattern Recognition (CVPR). (Las Vegas, NV, USA: IEEE)., 770–778, IEEE. doi: 10.1109/CVPR.2016.90

[B27] Huang H. Lin L. Tong R. Hu H. Zhang Q. Iwamoto Y.. (2020). “UNet 3+: A full-scale connected UNet for medical image segmentation,” in ICASSP 2020 - 2020 IEEE International Conference on Acoustics, Speech and Signal Processing (ICASSP), Barcelona, Spain: IEEE., 1055–1059. doi: 10.1109/ICASSP40776.2020.9053405

[B28] Ji M. Wu Z. (2022). Automatic detection and severity analysis of grape black measles disease based on deep learning and fuzzy logic. Comput. Electron Agric. 193, 106718. doi: 10.1016/J.COMPAG.2022.106718

[B29] Kukreja V. Baliyan A. Salonki V. Kaushal R. K. (2021). “Potato blight: deep learning model for binary and multi-classification,” in 2021 8th International Conference on Signal Processing and Integrated Networks (SPIN), Noida, India: IEEE, 967–672. doi: 10.1109/SPIN52536.2021.9566079

[B30] Kumar Y. Mishra A. (2023). “Few-shot referring relationships in videos,” in Proceedings of the IEEE/CVF Conference on Computer Vision and Pattern Recognition (CVPR). (Vancouver, Canada: Computer Vision Foundation (CVF)), 2289–2298.

[B31] Liang Q. Xiang S. Hu Y. Coppola G. Zhang D. Sun W. (2019). PD2SE-Net: Computer-assisted plant disease diagnosis and severity estimation network. Comput. Electron Agric. 157, 518–529. doi: 10.1016/J.COMPAG.2019.01.034

[B32] Liang X. (2021). Few-shot cotton leaf spots disease classification based on metric learning. Plant Methods 17, 1–11. doi: 10.1186/S13007-021-00813-7/FIGURES/13 34749780 PMC8576888

[B33] Li G. Wang Y. Zhao Q. Yuan P. Chang B. (2023). PMVT: a lightweight vision transformer for plant disease identification on mobile devices. Front. Plant Sci. 14. doi: 10.3389/fpls.2023.1256773 PMC10562605 37822342

[B34] Liu Z. Mao H. Wu C.-Y. Feichtenhofer C. Darrell T. Xie S. (2022). “A convNet for the 2020s,” in 2022 IEEE/CVF Conference on Computer Vision and Pattern Recognition (CVPR). (New Orleans, LA, USA: IEEE), 11966–11976. doi: 10.1109/CVPR52688.2022.01167

[B35] Ministry of Statistics & Programme Implementation (2023) Contribution of Agricultural Sector in GDP (Press Information Bureau). Available at: https://pib.gov.in/PressReleaseIframePage.aspx?PRID=1909213 (Accessed May 30, 2023).

[B36] Mwebaze E. Owomugisha G. (2016). “Machine learning for plant disease incidence and severity measurements from leaf images,” in 2016 15th IEEE International Conference on Machine Learning and Applications (ICMLA). (Anaheim, CA, USA: , IEEE), 158–163. doi: 10.1109/ICMLA.2016.0034

[B37] Nigam S. Jain R. Marwaha S. Arora A. Haque M. Dheeraj A.. (2023). Deep transfer learning model for disease identification in wheat crop. Ecol. Inform 75, 102068. doi: 10.1016/j.ecoinf.2023.102068

[B38] Pal A. Kumar V. (2023). AgriDet: Plant Leaf Disease severity classification using agriculture detection framework. Eng. Appl. Artif. Intell. 119, 105754. doi: 10.1016/j.engappai.2022.105754

[B39] Pan J. Xia L. Wu Q. Guo Y. Chen Y. Tian X. (2022). Automatic strawberry leaf scorch severity estimation via faster R-CNN and few-shot learning. Ecol. Inform 70, 101706. doi: 10.1016/j.ecoinf.2022.101706

[B40] Patil S. B. Bodhe S. K. (2011). Leaf disease severity measurement using image processing. Int. J. Eng. Technol. 3, 297–301.

[B41] Ramesh S. Hebbar R. M. N. R. P. N. P. B. N. S.. (2018). “Plant disease detection using machine learning,” in 2018 International Conference on Design Innovations for 3Cs Compute Communicate Control (ICDI3C). (Bangalore, India: IEEE), 41–45. doi: 10.1109/ICDI3C.2018.00017

[B42] Sandler M. Howard A. Zhu M. Zhmoginov A. Chen L.-C. (2018). “MobileNetV2: inverted residuals and linear bottlenecks,” in 2018 IEEE/CVF Conference on Computer Vision and Pattern Recognition. (Los Alamitos, CA, USA: IEEE Computer Society). 4510–4520. doi: 10.1109/CVPR.2018.00474

[B43] Sethy P. K. Negi B. Barpanda N. K. Behera S. K. Rath A. K. (2018). “Measurement of disease severity of rice crop using machine learning and computational intelligence,” in Cognitive Science and Artificial Intelligence. (Singapore: Springer), 1–11. doi: 10.1007/978-981-10-6698-6_1

[B44] Shewale M. V. Daruwala R. D. (2023). High performance deep learning architecture for early detection and classification of plant leaf disease. J. Agric. Food Res. 14, 100675. doi: 10.1016/j.jafr.2023.100675

[B45] Shu H. Liu J. Hua Y. Chen J. Zhang S. Su M.. (2023). A grape disease identification and severity estimation system. Multimed Tools Appl. 82, 23655–23672. doi: 10.1007/s11042-023-14755-w

[B46] Sujatha R. Chatterjee J. M. Jhanjhi N. Brohi S. N. (2021). Performance of deep learning vs machine learning in plant leaf disease detection. Microprocess Microsyst 80, 103615. doi: 10.1016/j.micpro.2020.103615

[B47] Szegedy C. Vanhoucke V. Ioffe S. Shlens J. Wojna Z. (2016). “Rethinking the inception architecture for computer vision,” in 2016 IEEE Conference on Computer Vision and Pattern Recognition (CVPR). (Las Vegas, NV, USA: IEEE), 2818–2826. doi: 10.1109/CVPR.2016.308

[B48] Szegedy C. Wei L. Yangqing J. Sermanet P. Reed S. Anguelov D.. (2015). “Going deeper with convolutions,” in 2015 IEEE Conference on Computer Vision and Pattern Recognition (CVPR). (Boston, MA, USA: IEEE), 1–9. doi: 10.1109/CVPR.2015.7298594

[B49] Tan M. Le Q. (2021). “EfficientNetV2: smaller models and faster training,” in Proceedings of the 38th International Conference on Machine Learning. Eds. Meila M. Zhang T. (Cambridge: Organized in Virtual Mode), 10096–10106.

[B50] Tassis L. M. Krohling R. A. (2022). Few-shot learning for biotic stress classification of coffee leaves. Artif. Intell. Agric. 6, 55–67. doi: 10.1016/j.aiia.2022.04.001

[B51] Tulshan A. S. Raul N. (2019). “Plant leaf disease detection using machine learning,” in 2019 10th International Conference on Computing, Communication and Networking Technologies (ICCCNT). (Kanpur, India: IEEE), 1–6. doi: 10.1109/ICCCNT45670.2019.8944556

[B52] Verma S. Chug A. Singh A. P. Singh D. (2023). PDS-MCNet: a hybrid framework using MobileNetV2 with SiLU6 activation function and capsule networks for disease severity estimation in plants. Neural Comput. Appl. 35, 18641–18664. doi: 10.1007/s00521-023-08693-9

[B53] Wang C. Du P. Wu H. Li J. Zhao C. Zhu H. (2021a). A cucumber leaf disease severity classification method based on the fusion of DeepLabV3+ and U-Net. Comput. Electron Agric. 189, 106373. doi: 10.1016/J.COMPAG.2021.106373

[B54] Wang G. Sun Y. Wang J. (2017). Automatic image-based plant disease severity estimation using deep learning. Comput. Intell. Neurosci. 2017, 1–8. doi: 10.1155/2017/2917536 PMC5516765 28757863

[B55] Wang Y. Yao Q. Kwok J. T. Ni L. M. (2021b). Generalizing from a few examples. ACM Comput. Surv 53, 1–34. doi: 10.1145/3386252

[B56] Wspanialy P. Moussa M. (2020). A detection and severity estimation system for generic diseases of tomato greenhouse plants. Comput. Electron Agric. 178, 105701. doi: 10.1016/J.COMPAG.2020.105701

[B57] Xing B. Wang D. Yin T. (2023). The evaluation of the grade of leaf disease in apple trees based on PCA-logistic regression analysis. Forests 14, 1290. doi: 10.3390/f14071290

[B58] Yang Q. Duan S. Wang L. (2022). Efficient identification of apple leaf diseases in the wild using convolutional neural networks. Agronomy 12, 2784. doi: 10.3390/agronomy12112784

[B59] Yang Q. Zhang Y. D. Pan W. Jialin S. (2020). “Few-shot learning,” in Transfer Learning (Cambridge University Press), 177–195. doi: 10.1017/9781139061773.015

[B60] Zhang Y. Wa S. Zhang L. Lv C. (2022). Automatic plant disease detection based on tranvolution detection network with GAN modules using leaf images. Front. Plant Sci. 13. doi: 10.3389/fpls.2022.875693 PMC9178295 35693164

[B61] Zhao R. Zhu Y. Li Y. (2023). CLA: A self-supervised contrastive learning method for leaf disease identification with domain adaptation. Comput. Electron Agric. 211, 107967. doi: 10.1016/j.compag.2023.107967

[B62] Zhao Y. Chen J. Xu X. Lei J. Zhou W. (2021). SEV‐Net: Residual network embedded with attention mechanism for plant disease severity detection. Concurr Comput. 33, 1–18. doi: 10.1002/cpe.6161

[B63] Zoph B. Vasudevan V. Shlens J. Le Q. V. (2018). “Learning transferable architectures for scalable image recognition,” in 2018 IEEE/CVF Conference on Computer Vision and Pattern Recognition. (Salt Lake City, UT, USA: IEEE), 8697–8710. doi: 10.1109/CVPR.2018.00907

