# Supplementary material for: PDSE-Lite: lightweight framework for plant disease severity estimation based on Convolutional Autoencoder and Few-Shot Learning
Source: Front Plant Sci. 2024 Jan 8;14:1319894. doi: 10.3389/fpls.2023.1319894 (PMC10800669; doi:10.3389/fpls.2023.1319894)
Supplement: Supplementary file 1 [file Table_1.docx]

Table S1: Implementation details of the CAE model

| **LayerNumber** | | **LayerName** | **InputShape** | **Connected to** | **OutputShape** | **Number of parameters** |
| --- | --- | --- | --- | --- | --- | --- |
| 1 | | Input Layer | (256, 256, 3) | - | (256, 256, 3) | 0 |
| 2 | Encoder block of CAE | Conv2D #1 | (256, 256, 3) | Input Layer | (256, 256, 16) | 448 |
| 3 |  | MaxPool2D #1 | (256, 256, 16) | Conv2D #1 | (128, 128, 16) | 0 |
| 4 |  | Conv2D #2 | (128, 128, 16) | MaxPool2D #1 | (128, 128, 8) | 1160 |
| 5 |  | MaxPool2D #2 | (128, 128, 8) | Conv2D #2 | (64, 64, 8) | 0 |
| 6 |  | Conv2D #3 | (64, 64, 8) | MaxPool2D #2 | (64, 64, 8) | 584 |
| 7 |  | MaxPool2D #3 | (64, 64, 8) | Conv2D #3 | (32, 32, 8) | 0 |
| 8 | Bottleneck layer of CAE | Conv2D #4 | (32, 32, 8) | MaxPool2D #3 | (32, 32, 8) | 584 |
| 9 | Decoder block of CAE | UpSample2D #1 | (32, 32, 8) | Conv2D #4 | (64, 64, 8) | 0 |
| 10 |  | Conv2D #5 | (64, 64, 8) | UpSample2D #1 | (64, 64, 8) | 584 |
| 11 |  | UpSample2D #2 | (64, 64, 8) | Conv2D #5 | (128, 128, 8) | 0 |
| 12 |  | Conv2D #6 | (128, 128, 8) | UpSample2D #2 | (128, 128, 8) | 584 |
| 13 |  | UpSample2D #3 | (128, 128, 8) | Conv2D #6 | (256, 256, 8) | 0 |
| 14 | Output Layer of CAE | Conv2D #7 | (256, 256, 8) | UpSample2D #3 | (256, 256, 3) | 219 |
| **Total weight parameters** | | | | | | 4163 |
