# Supplementary material for: PDSE-Lite: lightweight framework for plant disease severity estimation based on Convolutional Autoencoder and Few-Shot Learning
Source: Front Plant Sci. 2024 Jan 8;14:1319894. doi: 10.3389/fpls.2023.1319894 (PMC10800669; doi:10.3389/fpls.2023.1319894)
Supplement: Supplementary file 3 [file Table_3.docx]

Table S3: Layer-wise implementation details of the PDSE-Lite framework’s image segmentation model used to segment diseased areas from leaf images

| **LayerNumber** | | **LayerName** | **InputShape** | **Connected to** | **OutputShape** | **Number of parameters** |
| --- | --- | --- | --- | --- | --- | --- |
| 1 | | Input Layer | (256, 256, 3) | - | (256, 256, 3) | 0 |
| 2 | Layers from the Encoder block of CAE | Conv2D #1 | (256, 256, 3) | Input Layer | (256, 256, 16) | 448 |
| 3 |  | MaxPool2D #1 | (256, 256, 16) | Conv2D #1 | (128, 128, 16) | 0 |
| 4 |  | Conv2D #2 | (128, 128, 16) | MaxPool2D #1 | (128, 128, 8) | 1160 |
| 5 |  | MaxPool2D #2 | (128, 128, 8) | Conv2D #2 | (64, 64, 8) | 0 |
| 6 |  | Conv2D #3 | (64, 64, 8) | MaxPool2D #2 | (64, 64, 8) | 584 |
| 7 |  | MaxPool2D #3 | (64, 64, 8) | Conv2D #3 | (32, 32, 8) | 0 |
| 8 | Bottleneck layer of CAE | Conv2D #4 | (32, 32, 8) | MaxPool2D #3 | (32, 32, 8) | 584 |
| 9 | Layers from the Decoder block of CAE | UpSample2D #1 | (32, 32, 8) | Conv2D #4 | (64, 64, 8) | 0 |
| 10 |  | Conv2D #5 | (64, 64, 8) | UpSample2D #1 | (64, 64, 8) | 584 |
| 11 |  | UpSample2D #2 | (64, 64, 8) | Conv2D #5 | (128, 128, 8) | 0 |
| 12 |  | Conv2D #6 | (128, 128, 8) | UpSample2D #2 | (128, 128, 8) | 584 |
| 13 | Extra Layers added to the image segmentation model | UpSample2D #4 | (32, 32, 8) | Conv2D #4 | (256, 256, 8) | 0 |
| 14 |  | UpSample2D #5 | (64, 64, 8) | Conv2D #5 | (256, 256, 8) | 0 |
| 15 |  | UpSample2D #6 | (128, 128, 8) | Conv2D #6 | (256, 256, 8) | 0 |
| 16 |  | Concatenate | (256, 256, 8)  (256, 256, 8)  (256, 256, 8) | UpSample2D #4  UpSample2D #5  UpSample2D #6 | (256, 256, 24) | 0 |
| 17 |  | Conv2D #10 | (256, 256, 24) | Concatenate | (256, 256, 12) | 2604 |
| 18 |  | Conv2D #11 | (256, 256, 12) | Conv2D #10 | (256, 256, 6) | 654 |
| 19 |  | Conv2D #12 (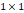 filter size) | (256, 256, 6) | Conv2D #11 | (256, 256, 3) | 21 |
| **Total weight parameters** | | | | | | 7223 |
| **Total non-trainable weight parameters** | | | | | | 3944 |
| **Total trainable weight parameters** | | | | | | 3279 |
