# Supplementary material for: PDSE-Lite: lightweight framework for plant disease severity estimation based on Convolutional Autoencoder and Few-Shot Learning
Source: Front Plant Sci. 2024 Jan 8;14:1319894. doi: 10.3389/fpls.2023.1319894 (PMC10800669; doi:10.3389/fpls.2023.1319894)
Supplement: Supplementary file 4 [file Table_4.docx]

Table S4: Details of leaf images present in training, validation, and testing subsets

| **Class/ Type of**  **Leaf image** | **Number of Instances** | | |
| --- | --- | --- | --- |
|  | **Training**  **Subset** | **Validation**  **Subset** | **Test Subset** |
| Alternaria Leaf Spot | 194 | 42 | 42 |
| Brown Spot | 150 | 32 | 33 |
| Gray Spot | 276 | 59 | 60 |
| Healthy | 286 | 61 | 62 |
| Rust | 240 | 52 | 52 |
| **Total** | **1146** | **246** | **249** |
